# Efficacy and safety of fecal microbiota transplantation for the treatment of diseases other than *Clostridium difficile* infection: a systematic review and meta-analysis

**DOI:** 10.1080/19490976.2020.1854640

**Published:** 2020-12-19

**Authors:** Jessica Emily Green, Jessica A. Davis, Michael Berk, Christopher Hair, Amy Loughman, David Castle, Eugene Athan, Andrew A. Nierenberg, John F. Cryan, Felice Jacka, Wolfgang Marx

**Affiliations:** a IMPACT, the Institute for Mental and Physical Health and Clinical Translation, Food & Mood Centre, School of Medicine, Barwon Health, Deakin University, Geelong, Australia; b Monash Alfred Psychiatry Research Centre (Maprc), Central Clinical School, Faculty of Medicine Nursing and Health Sciences, Monash University, Melbourne, Australia; c Department of Psychiatry, Peninsula Health, Frankston, Australia; d Department of Psychiatry, University of Melbourne, Parkville, Australia; e Orygen Youth Health Research Centre and the Centre of Youth Mental Health, Melbourne, Australia; f The Florey Institute for Neuroscience and Mental Health, Parkville, Australia; g Barwon Health, Geelong, Australia; h Department of Psychiatry, St Vincent’s Health, East Melbourne, Australia; i School of Medicine, Deakin University, Geelong, Australia; j Department of Psychiatry, Dauten Family Center for Bipolar Treatment Innovation, Boston, MA, USA; k Harvard Medical School, Boston, MA, USA; l Department of Anatomy and Neuroscience, University College Cork and APC Microbiome, Ireland; m Centre for Adolescent Health, Murdoch Children’s Research Institute, Royal Children’s Hospital, Parkville, Australia; n Black Dog Institute, Melbourne, Australia; o James Cook University, Townsville, Australia

**Keywords:** Fecal microbiota transplantation clostridium difficile, microbiome, meta-analysis, RCT, systematic review, ulcerative colitis, irritable bowel syndrome, psychiatry, mental disorder, neuroscience, depression

## Abstract

The intestinal microbiome has been identified as a key modifier for a variety of health conditions. Fecal Microbiota Transplantation (FMT) has emerged as a fast, safe, and effective means by which to modify the intestinal microbiome and potentially treat a variety of health conditions. Despite extensive research of FMT for CDI, there is a lack of clarity informed by systematic synthesis of data regarding the safety and efficacy of FMT for other health conditions. This systematic review used PRISMA guidelines and was prospectively registered with PROSPERO (CRD42018104243). In March 2020, a search of MEDLINE, EMBASE, and PsycINFO was conducted. We identified 26 eligible studies. A meta-analysis of FMT for active Ulcerative Colitis (UC) showed that FMT significantly improved rates of clinical remission (OR = 3.634, 95% CI = 1.940 to 6.808, I^2^ = 0%, *p* < .001), clinical response (OR = 2.634, 95% CI = 1.441 to 4.815, I^2^ = 33%, *p* = .002) and endoscopic remission (OR = 4.431, 95% CI = 1.901 to 10.324, I^2^ = 0%, *p* = .001). With respect to Irritable Bowel Syndrome, a meta-analysis showed no significant change in symptoms following FMT (*p* = .739). Hepatic disorders, metabolic syndrome, and antibiotic-resistant organisms were conditions with emerging data on FMT. Serious adverse events (AE) were more often reported in control group participants (n = 43) compared with FMT group participants (n = 26). There were similar rates of mild to moderate AE in both groups. Preliminary data suggest that FMT is a potentially safe, well-tolerated and efficacious treatment for certain conditions other than CDI, with evidence for active UC being the most compelling.

## Introduction

The intestinal microbiome has emerged as a modifiable target for treating a variety of health conditions thought to be associated with dysregulated microbiome profiles.^1^ The intestinal microbiome is believed to have a key role in modifying immunity, inflammation, and – by extension – a plethora of health conditions.^2–4^ There is now substantial research interest^5^ into interventions that might target the gut microbiome to improve chronic diseases, including diet, supplementary prebiotics, probiotics, antibiotics, short-chain fatty acids, and Fecal Microbiota Transplantation (FMT).^6,7^

FMT is a technique in which gut bacteria are transferred from a healthy donor to a patient, with the goal of introducing or restoring a stable microbial community in the gut. FMT has been established as an effective means of rapidly modifying the intestinal microbiota and may therefore have potential as a treatment for the many health conditions linked with the intestinal microbiome.^8^ FMT is already widely practiced as a highly effective treatment for recurrent *Clostridium difficile* infection (CDI).^9,10^

A wealth of new research is investigating whether FMT may be used to treat other health conditions linked to the intestinal microbiome,^11,12^ including gastrointestinal,^13–17^ autoimmune,^18,19^ metabolic,^20,21^ and neuropsychiatric^22–24^ conditions. There is also promising preclinical evidence supporting the use of FMT in conditions other than CDI, including Major Depressive Disorder,^25,26^ schizophrenia,^27^ and cardiometabolic syndrome.^28^

While reviews of FMT for specific indications such as IBS^29–31^ and IBD exist,^32–34^ to date there have been no comprehensive reviews evaluating and synthesizing the entire body of data for both the efficacy and safety of FMT for all conditions other than CDI. This systematic review and meta-analysis addresses the question of whether FMT is safe and effective at treating health conditions other than CDI in humans.

## Methods

### Protocol and registration

The Preferred Reporting Items for Systematic Reviews and Meta-Analyses (PRISMA) guidelines were adhered to as a methodological template for this review. The protocol for this Systematic Review was prospectively registered with PROSPERO (CRD42018104243).

### Search strategy and eligibility criteria

The PICO approach (population, intervention, comparator, outcomes) was used to guide the search strategy for this review. The PICO criteria used are outlined below:

- Population: Humans participants of any age with any acute or chronic health condition other than CDI. Studies were included only if participants were followed up for at least two weeks post-FMT.
- Intervention: All possible variations of human FMT were included. For the purposes of this review, FMT was defined as any process by which a fecal microbiota suspension was transferred from the gastrointestinal tract of a healthy individual into another person with the aim of treating a health condition.^35^
- Comparator: Studies were included if they utilized a control group.
- Outcomes: When reporting on efficacy, this review used primary outcome measures as described by each study. When the primary outcome did not relate to efficacy, the secondary outcomes relating to clinical efficacy were noted, but results were only considered significant when the primary outcome measure related to clinical efficacy and was statistically significant vs the control intervention. Adverse events (AE) were reported as presented by the included study.

In March 2020, searches were carried out using MEDLINE, EMBASE, Cochrane Central Register of Controlled Trials, Health Technology Assessment Database, Allied and Complementary Medicine (AMED) and PsycINFO. Reference sections of previously published randomized trials, systematic reviews, and meta-analyses on this and related topics were also searched.

Forty-two iterations of the term “FMT” were identified and used as search terms:

FMT or fecal microbiota transplant* or fecal microbiota transplant* or microbiota transfer or microbiome transfer or microbiota transplant* OR microbiome transplant* or microbial transplant* or microbial transfer or fecal transplant* OR fecal transplant* or feces transplant* OR feces transplant* or stool transplant* or stool transfer or fecal flora transplant* OR fecal flora transplant* or microflora transplant* OR fecal flora transfer or fecal flora transfer OR fecal bacteriotherapy OR fecal bacteriotherapy OR feces bacteriotherapy OR feces bacteriotherapy OR rectal bacteriotherapy OR fecal flora bacteriotherapy OR donor fecal OR donor stool OR donor feces OR donor fecal or donor feces fecal transfer OR fecal transfer OR fecal reconstitution OR fecal reconstitution OR flora reconstitution OR microbiome reconstitution OR feces reconstitution or feces reconstitution. The following modifiers were applied: studies relating to humans and published in English.

### Study selection

The following study types were included: randomized controlled trials (RCTs), non-randomized-controlled studies, and observational studies with a comparator arm. In the case of observational studies with a comparator arm, only prospective cohort studies were included in order to assess temporality. Reviews, abstracts, conference papers, and posters were excluded.

Two investigators (JG and JD) independently performed the searches using Rayyan software. JG performed initial screening to identify potentially eligible studies. Articles were first screened by title and abstract. Remaining articles were further scrutinized by full-text review. JD acted as a secondary reviewer and was blind to JG’s screening outcomes. Where there was a lack of consensus between the two reviewers, the senior author (WM) acted as a third reviewer to make a final decision on whether the study met inclusion criteria.

#### Risk of bias assessment

Methodological heterogeneity was evaluated by comparing included data using the ‘risk of bias’ tables. The Cochrane Risk of Bias tool was used to assess the risk of bias in randomized trials. JG and JD independently evaluated risk of bias.^36^

### Statistical analysis

Data from individual trials were to be combined, and a meta-analysis performed only if the data were amenable. Patient groups, disease entity, and outcome measures needed to be sufficiently similar in order for synthesis to occur. Our meta-analyses were conducted in Comprehensive Meta-Analysis (version 3.3.070)^37^ using a Mantel-Haenszel random-effects model to account for heterogeneity between studies. The I^2^-statistic was used as an indicator of heterogeneity. A value of 0% indicates no observed heterogeneity, and larger values indicate increasing heterogeneity. Due to the limited number of studies included in each meta-analysis, no sensitivity or subgroup analyses were performed. Furthermore, no test of publication bias was performed due to the limited number of trials.^38^

### Assessment of microbial “engraftment”

This review also assessed whether successful “engraftment” occurred of the donor microbiome in the recipient. For the purposes of this review, the term “engraftment” was ascertained according to the following key concepts:

- Was a change in recipient microbiota observed following FMT?

If a change in recipient microbiota was observed, then:

- Was this change toward the donor microbiota?
- To what extent/how significant was that change?
- For how long did the microbiota changes persist following cessation of FMT?

## Results

### Study selection

**Figure 1.**
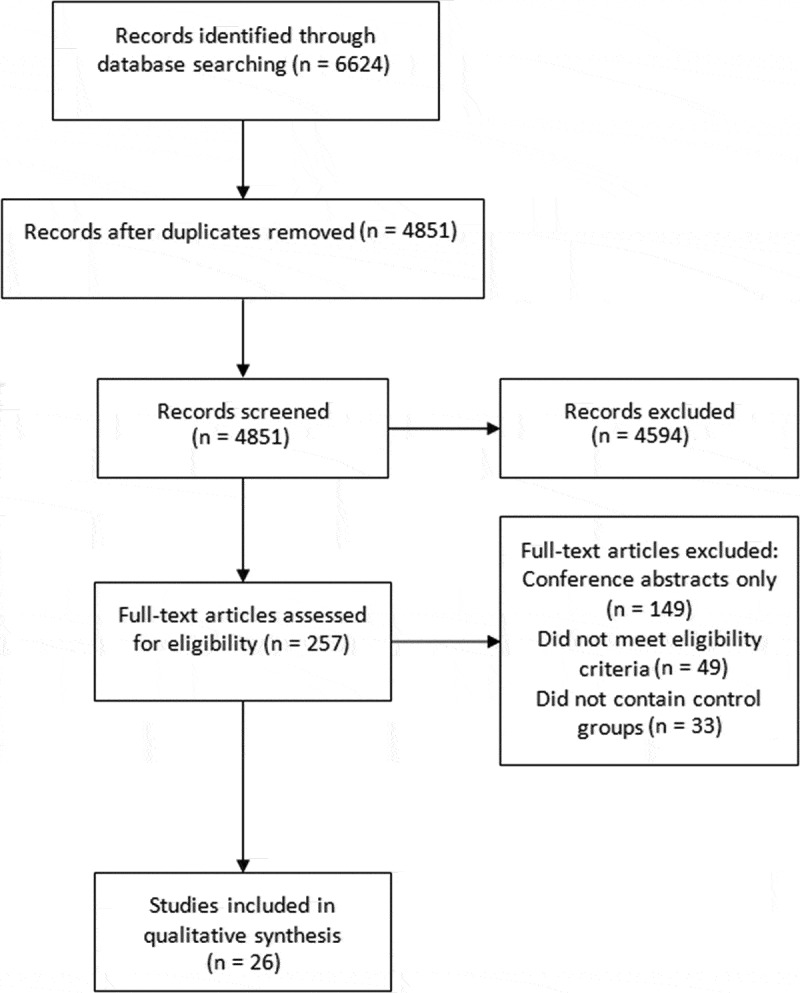
PRISMA flowchart of included studies

The systematic search identified 5,495 de-duplicated studies, of which 26 met eligibility criteria for inclusion (see Figure 1).

### Study characteristics

As per Table 1, of the 26 articles that were included in the final review, 20 were Randomized Controlled Trials (RCTs), two of which were open-label, three single-blind and 15 double-blind. The remaining six studies encompassed non-randomized-controlled studies (n = 3), case–control studies (n = 2), and cohort studies (n = 1). Eight studies investigated the use of FMT for Inflammatory Bowel Disease (IBD), six for functional gut disorders, four for hepatic disorders, three for metabolic syndrome, two for antibiotic-resistant organisms, and one each for “pouchitis,” obesity without metabolic syndrome, and Human Immunodeficiency Virus (HIV).

**Table 1.** Summary of FMT methodological factors

Study details	Methodological factors	Dosing regimen (ie. dosage, frequency, duration)	Donor factors	Adjunctive treatment	Control intervention
Saidani et al, 2019	Route of administration: NGT Aerobic vs. anaerobic preparation: aerobic Fresh vs. frozen: fresh	Duration of intervention: once-off Initial dosage: 50 g stool Maintenance regimen: N/A	Number of donors (i.e. single vs. multiple): not stated Type of donor (e.g. Relative vs. non-related): not stated Donor screening for mental illness or metabolic risk factors: Unclear, stated used “French authorities’ recommendations”	Antibiotic pre-treatment of recipients: yes (colistin 6-MUI and aminoglycoside (either gentamycin or amikacin) if sensitive, or other antibiotics if resistant Bowel cleanse: bowel preparation 5 days prior, and day before FMT Other: nasopharangeal decolonization using chlorhexidine gluconate gargle and nose swab, and pantoprazole	Description of control intervention: treatment as usual
Huttner et al, 2019	Route of administration: NGT (two sites – Israel and The Netherlands) or encapsulated FMT (two sites – Switzerland and France) Aerobic vs. anaerobic preparation: aerobic Fresh vs. frozen: frozen	Duration of intervention: NGT as once off, followed by daily encapsulated FMT for 2 consecutive days Initial dosage: 40 g stool via NGT, unclear dosing for encapsulated FMT Maintenance regimen: N/A	Number of donors (i.e. single vs. multiple):single Type of donor (e.g. Relative vs. non-related): not related Donor screening for mental illness or metabolic risk factors: Screened for “chronic diseases or medical history” but not mental health or metabolic risk factors specifically	Antibiotic pre-treatment of recipients: yes (colistin sulfate and neomycin sulfate) Bowel cleanse: no Other: N/A	Description of control intervention: treatment as usual
Sokol et al, 2020	Route of administration: colonoscopy Aerobic vs. anaerobic preparation: aerobic Fresh vs. frozen: fresh	Duration of intervention: once-off Initial dosage: 50–100 g fresh stool Maintenance regimen: N/A	Number of donors (i.e. single vs. multiple):single Type of donor (e.g. Relative vs. non-related):non-related Donor screening for mental illness or metabolic risk factors: states excluded if “chronic disease, long term treatment” or BMI > 27, but screening for mental illness or risk factors not specifically described.	Antibiotic pre-treatment of recipients: no Bowel cleanse: bowel preparation with polyethylene glycol Other: N/A	Description of control intervention: same but placebo (physiological serum) used in sham group
Bajaj et al, 2017	Route of administration: retention enema Aerobic vs. anaerobic preparation: aerobic Fresh vs. frozen: frozen	Duration of intervention: once-off Initial dosage: 90 mL frozen aliquot of fecal suspension Maintenance regimen: N/A	Number of donors (i.e. single vs. multiple): single Type of donor (e.g. Relative vs. non-related): non-related Donor screening for mental illness or metabolic risk factors: metabolic risk factors – yes mental illness – yes	Antibiotic pre-treatment of recipients:yes (metronidazole, ciprofloxacin, amoxicillin) Bowel cleanse: no Other: N/A	Description of control intervention: treatment as usual
Bajaj et al, 2019	Route of administration: encapsulated FMT Aerobic vs. anaerobic preparation: aerobic Fresh vs. frozen: frozen	Duration of intervention: once-off Initial dosage: 15 capsules (unclear dosage per capsule) Maintenance regimen: N/A	Number of donors (i.e. single vs. multiple): single Type of donor (e.g. Relative vs. non-related): non-related Donor screening for mental illness or metabolic risk factors: metabolic risk factors – yes mental illness – yes	Antibiotic pre-treatment of recipients: no Bowel cleanse: no Other: N/A	Description of control intervention: same but placebo capsules used (containing a sterile solution of cocoa butter, glycerol and brown food color).
Aroniadis et al, 2019	Route of administration: encapsulated FMT Aerobic vs. anaerobic preparation: Not specified Fresh vs. frozen: frozen	Duration of intervention: 3 days Initial dosage: 25 capsules containing 9.5 g fresh stool each Maintenance regimen: two further doses of 25 capsules over the next 2 days	Number of donors (i.e. single vs. multiple): single Type of donor (e.g. Relative vs. non-related): non-related Donor screening for mental illness or metabolic risk factors: metabolic risk factors – yes mental illness – yes	Antibiotic pre-treatment of recipients: no Bowel cleanse: no Other: esomeprazole	Description of control intervention: same, but placebo capsules used (containing nontoxic brown pigment)
El-Salhy et al, 2019	Route of administration: NDT Aerobic vs. anaerobic preparation: aerobic Fresh vs. frozen: frozen	Duration of intervention: once-off Initial dosage: 30 or 60 g feces Maintenance regimen: N/A	Number of donors (i.e. single vs. multiple): single Type of donor (e.g. Relative vs. non-related): non-related Donor screening for mental illness or metabolic risk factors: metabolic risk factors – screened for “metabolic disorders” mental illness – no	Antibiotic pre-treatment of recipients: no Bowel cleanse: no Other: N/A	Description of control intervention: same but autologous FMT used
Holster et al, 2019	Route of administration: colonoscopy Aerobic vs. anaerobic preparation: aerobic Fresh vs. frozen: frozen	Duration of intervention: once-off Initial dosage: 30 g feces Maintenance regimen: N/A	Number of donors (i.e. single vs. multiple):single Type of donor (e.g. Relative vs. non-related): non-related Donor screening for mental illness or metabolic risk factors: metabolic risk factors – yes mental illness – yes	Antibiotic pre-treatment of recipients: no Bowel cleanse: bowel preparation prior to procedure Other: N/A	Description of control intervention: same but autologous FMT used
Vrieze et al, 2012	Route of administration: NDT Aerobic vs. anaerobic preparation: aerobic Fresh vs. frozen: fresh	Duration of intervention: once-off Initial dosage: 500 mL aliquot of FMT suspension Maintenance regimen: N/A	Number of donors (i.e. single vs. multiple): single Type of donor (e.g. Relative vs. non-related): non-related Donor screening for mental illness or metabolic risk factors: metabolic risk factors – screened for diabetes and obesity, but did not specify other metabolic risk factors mental illness – no	Antibiotic pre-treatment of recipients: no Bowel cleanse: bowel lavage the night before Other: fasting the night before	Description of control intervention: same but autologous FMT used
Smits et al, 2019	Route of administration: NDT Aerobic vs. anaerobic preparation: aerobic Fresh vs. frozen: fresh	Duration of intervention: once-off Initial dosage: 500 mL aliquot of FMT suspension Maintenance regimen: N/A	Number of donors (i.e. single vs. multiple): single Type of donor (e.g. Relative vs. non-related): non-related Donor screening for mental illness or metabolic risk factors: metabolic risk factors – yes mental illness – no	Antibiotic pre-treatment of recipients: no Bowel cleanse: bowel lavage Other: N/A	Description of control intervention: same but autologous FMT used
Allegretti et al, 2019	Route of administration: encapsulated FMT Aerobic vs. anaerobic preparation: aerobic Fresh vs. frozen: frozen	Duration of intervention: 8 weeks (3 doses given 4 weeks apart) Initial dosage: 30 capsules, containing 0.75 g stool per capsule Maintenance regimen: 12 capsules given at 4 and 8 weeks	Number of donors (i.e. single vs. multiple): single Type of donor (e.g. Relative vs. non-related):non-related Donor screening for mental illness or metabolic risk factors: metabolic risk factors – yes mental illness – yes	Antibiotic pre-treatment of recipients: no Bowel cleanse: no Other: N/A	Description of control intervention: same but placebo capsules used, containing normal saline, food color and glycerol)
Costello et al, 2019	Route of administration: colonoscopy followed by retention enemas Aerobic vs. anaerobic preparation: anaerobic Fresh vs. frozen: frozen	Duration of intervention: 3 doses given in 1 week Initial dosage: 50 g stool Maintenance regimen: two enemas containing 25 g stool within first week	Number of donors (i.e. single vs. multiple): pooled (3–4 donors, mixed) Type of donor (e.g. Relative vs. non-related): non-related Donor screening for mental illness or metabolic risk factors: metabolic risk factors – yes mental illness – screened for depression, but not other mental illnesses	Antibiotic pre-treatment of recipients: no Bowel cleanse: bowel preparation with polyethylene glycol the night before Other: loperimide immediately prior to procedure	Description of control intervention: same but autologous FMT used
Sood et al, 2019	Route of administration: colonoscopy Aerobic vs. anaerobic preparation: aerobic Fresh vs. frozen: frozen	Duration of intervention: 42 weeks (6 doses, 8 weeks apart) Initial dosage: 100 g feces Maintenance regimen: same	Number of donors (i.e. single vs. multiple): single Type of donor (e.g. Relative vs. non-related): non-related Donor screening for mental illness or metabolic risk factors: metabolic risk factors – no mental illness – no	Antibiotic pre-treatment of recipients: no Bowel cleanse: polyethylene glycol bowel lavage the night before Other: N/A	Description of control intervention: same but placebo FMT (normal saline with food color) used
Herfarth et al, 2019	Route of administration: endoscopic route (unspecified whether NGT, NDT or NJT), followed by encapsulated FMT Aerobic vs. anaerobic preparation: not specified Fresh vs. frozen: not specified	Duration of intervention: 14 days (endoscopic FMT, followed by daily encapsulated FMT) Initial dosage: 24 g stool Maintenance regimen: 6 capsules daily for 14 days (total dose 4.2 g stool/day)	Number of donors (i.e. single vs. multiple): single Type of donor (e.g. Relative vs. non-related): non-related Donor screening for mental illness or metabolic risk factors: metabolic risk factors – Screened for BMI but unclear if screened for other metabolic risk factors mental illness – yes	Antibiotic pre-treatment of recipients: not specified Bowel cleanse: not specified Other: not specified	Description of control intervention: same but inert placebo FMT used
Philips et al, 2018^14^	Route of administration: NDT Aerobic vs. anaerobic preparation: aerobic Fresh vs. frozen: fresh	Duration of intervention: daily for 7 days Initial dosage: 100 mL of strained and filtered stool Maintenance regimen: same	Number of donors (i.e. single vs. multiple): single Type of donor (e.g. Relative vs. non-related): related Donor screening for mental illness or metabolic risk factors: metabolic risk factors – yes, mental illness – no	Antibiotic pre-treatment of recipients: antibiotics were continued if the person was already on them Bowel cleanse: no Other: fasting 4 hours before and after	Description of control intervention: control groups consisted of alternative treatments (steroids, nutrition or pentoxifylline therapy)
Ishikawa et al, 2017^39^	Route of administration: colonoscopy Aerobic vs. anaerobic preparation: aerobic Fresh vs. frozen: fresh	Duration of intervention: once off Initial dosage: 150–250 g fresh donor stool Maintenance regime: N/A	Number of donors (ie. single vs. multiple): single Type of donor (eg. Relative vs. non-related): related (spouses or relative) Donor screening for mental illness or metabolic risk factors: not comprehensively described – states “medical history”	Antibiotic pre-treatment of recipients: yes (amoxicillin, fosfomycinand metronidazole) Bowel cleanse: bowel preparation given, in addition to bowel lavage with poloyethylene glycol given prior to treatment Other: Scopolamine given post-treatment to slow gastric transit time	Description of control intervention: 2 weeks antibiotics only (Amoxicillin, Fosfomycin, metronidazole)
Tian et al, 2017^40^	Route of administration: NJT Aerobic vs. anaerobic preparation: aerobic Fresh vs. frozen: frozen	Duration of intervention: daily for 6 days Initial dosage: 100 mL fresh stool Maintenance regime: same	Number of donors (ie. single vs multiple): single Type of donor (eg. Relative vs. non-related): non-related Donor screening for mental illness or metabolic risk factors: screened for metabolic syndrome and “any ongoing diseases”	Antibiotic pre-treatment of recipients: no Bowel cleanse: nil specified Other: N/A	Description of control intervention: Rx as usual (12 weeks of education, behavioral strategies and oral laxatives), avoidance of probiotics.
Vujkovic-Cvijin et al, 2017^30^	Route of administration: colonoscopy Aerobic vs. anaerobic preparation: aerobic Fresh vs. frozen: frozen	Duration of intervention: once-off Initial dosage: 250 mL of stool suspension Maintenance regime: N/A	Number of donors (ie. single vs. multiple): single Type of donor (eg. Relative vs. non-related): non-related Donor screening for mental illness or metabolic risk factors: yes (Openbiome protocol used)	Antibiotic pre-treatment of recipients: no Bowel cleanse: bowel preparation used (Golytely) Other: N/A	Description of control intervention: treatment as usual
Johnsen et al, 2017^41^	Route of administration: colonoscopy Aerobic vs. anaerobic preparation: aerobic Fresh vs. frozen: fresh or frozen (1:1 ratio)	Duration of intervention: once-off Initial dosage: not described Maintenance regime: same Frequency of doses aerobic	Number of donors (ie. single vs. multiple): pooled (2 donors) and these were mixed. Type of donor (eg. Relative vs. non-related): non-related Donor screening for mental illness or metabolic risk factors: screened for metabolic syndrome, and chronic fatigue but not specifically for mental illness	Antibiotic pre-treatment of recipients: no Bowel cleanse: Picoprep bowel preparation used Other: loperamide used prior to procedure	Description of control intervention:same but with autologous FMT.
Kootte et al, 2017^36^	Route of administration: NDT Aerobic vs. anaerobic preparation: partially anaerobic Fresh vs. frozen: fresh	Duration of intervention: once-off Initial dosage: not clearly described Maintenance regime: N/A	Number of donors (ie. single vs. multiple): single Type of donor (eg. Relative vs. non-related): non-related Donor screening for mental illness or metabolic risk factors: not screened for mental illness but screened for metabolic risk factors	Antibiotic pre-treatment of recipients: no Bowel cleanse: bowel lavage prior to procedure Other: fasting prior to procedure	Description of control intervention: same plus autologous FMT prepared in same way. Taken 6 hours prior to FMT
Rossen et al, 2015^37^	Route of administration: NDT Aerobic vs. anaerobic preparation: aerobic Fresh vs. frozen: fresh	Duration of intervention: two FMTs given 3 weeks apart Initial dosage: 120 g fresh stool (average) Maintenance regime: same	Number of donors (ie. single vs. multiple): single, except for 6 recipients Type of donor (eg. Relative vs. non-related): non-related Donor screening for mental illness or metabolic risk factors: no	Antibiotic pre-treatment of recipients: no Bowel cleanse: bowel preparation (Moviprep) evening before and morning of FMT Other: N/A	Description of control intervention: same plus autologous FMT prepared in same way. Taken on morning of FMT
Kump et al, 2017^42^	Route of administration: colonoscopy (for 1^st^ FMT), and via sigmoidoscopy for subsequent FMTs Aerobic vs. anaerobic preparation: aerobic Fresh vs. frozen: fresh	Duration of intervention: 5 FMTs given 14 days apart (8 weeks total duration) Initial dosage: 250–500 mL fecal suspension Maintenance regime: same	Number of donors (ie. single vs. multiple): single Type of donor (eg. Relative vs. non-related): 6 donors were related, 2 were partners and 3 were relatives Donor screening for mental illness or metabolic risk factors: screened for BMI but no other metabolic risk factors or mental illness.	Antibiotic pre-treatment of recipients: yes (vancomycin, paromomycin and nystatin) Bowel cleanse: bowel preparation (Moviprep) given prior to first FMT but not subsequent FMTs Other: N/A	Description of control intervention: antibiotics only
Moayyedi et al, 2015^43^	Route of administration: retention enema Aerobic vs. anaerobic preparation: aerobic Fresh vs. frozen: fresh or frozen (method of selection not described)	Duration of intervention: weekly for 6 weeks Initial dosage: 50 g of fresh stool in fecal suspension Maintenance regime: same	Number of donors (ie. single vs. multiple): single Type of donor (eg. Relative vs. non-related): not specified Donor screening for mental illness or metabolic risk factors: not clearly described	Antibiotic pre-treatment of recipients: no Bowel cleanse: no Other: N/A	Description of control intervention: same but with placebo (water enema)
Paramsothy et al, 2017^44^	Route of administration: colonoscopy (1^st^ treatment) colonoscopically followed by self-administered enemas) Aerobic vs. anaerobic preparation: aerobic Fresh vs. frozen: fresh	Duration of intervention: 8 weeks Initial dosage: 37.5 g fresh stool in 150 mL of suspension Maintenance regime: 5 enemas/week for 8 weeks. Same dosage	Number of donors (ie. single vs. multiple): multiple (3–7 donors, and these were mixed) Type of donor (eg. Relative vs. non-related): non-related Donor screening for mental illness or metabolic risk factors: yes – for metabolic syndrome, not specifically for mental illness but did screen for chronic pain syndrome and neurologic/neurodevelopmental disorders	Antibiotic pre-treatment of recipients: no Bowel cleanse: bowel preparation used prior to procedure Other: N/A	Description of control intervention: same but with placebo enema (isotonic saline, brown food colorouant, odourant and glycerol cryoprotectant 10%
Halkjaer et al, 2018^45^	Route of administration: orally (capsules) Aerobic vs. anaerobic preparation: partially anaerobic Fresh vs. frozen: frozen	Duration of intervention: daily for 12 days Initial dosage: 25 capsules (12 g frozen fecal matter derived from 50 g fresh feces) Maintenance regime: same	Number of donors (ie. single vs. multiple): multiple (4 donors) Type of donor (eg. Relative vs. non-related): not specified Donor screening for mental illness or metabolic risk factors: excluded if family history of GI diseases, cancer, diabetes, obesity, autoimmune diseases, allergy, asthma, eczema, cardiovascular diseases, neurologic or mental illnesses; however, does not describe if excluded if past hx of these diseases	Antibiotic pre-treatment of recipients: no Bowel cleanse: bowel preparation (picoprep) given prior to procedure Other: fasting prior to procedure	Description of control intervention: placebo capsules containing saline, 30% glycerol and food coloring (E150)
Ren et al, 2017^46^	Route of administration: NDT Aerobic vs. anaerobic preparation: aerobic Fresh vs. frozen: not described	Duration of intervention: every 4 weeks for 1–7 treatments Initial dosage: 80 mL of bacterial suspension Maintenance regime: same	Number of donors (ie. single vs. multiple): single Type of donor (eg. Relative vs. non-related): not specified Donor screening for mental illness or metabolic risk factors: not described	Antibiotic pre-treatment of recipients: participants were already on Hep B treatment, but nil specific pre-treatment described Bowel cleanse: not described Other: not described	Description of control intervention: treatment as usual

A total of 1149 participants were enrolled in the included studies, with a mean of 44 participants per study (sample sizes ranged from 6 to 165). When broken down by disorder, there were a total of 463 participants in studies relating to IBD, 424 for functional gut disorders, 104 for metabolic syndrome/obesity, 109 for hepatic disorders, 60 for antibiotic-resistant organisms, and 14 for other disorders. Study follow-up periods varied from 2 weeks to 12 months. The largest sub-groups by disorder were UC (n = 6) and irritable bowel syndrome (IBS) (n = 5). These groups were large enough to allow for meta-analyses.

Seventeen studies included both males and females, eight included males only, and one did not provide demographic data. Studies were conducted in US (n = 6),^41,44,47–51^ The Netherlands (n = 4),^39,42,43,52^ Australia (n = 2),^53,54^ China (n = 2), Norway (n = 2),^14,40^ France (n = 2),^45,46^ India (n = 2), Denmark (n = 1), Japan (n = 1),^55^ Austria (n = 1),^56^ Canada (n = 1),^57^ Sweden (n = 1),^58^ and one was an international multi-site collaborative study between Switzerland, the Netherlands, Israel, and France.^59^

### Methodological factors for FMT manufacture process

The FMT manufacture process varied significantly between studies (see Table 1); indeed, no two studies used the same process. Of the 26 included FMT studies, 11 delivered FMT via colonoscopy, enema, or both,^14,45,47,48,53–58,60^ nine were delivered endoscopically, either via nasojejunal tube,^61^nasoduodenal tube^39,40,42,43,52,62,63^ or nasogastric tube;^46^ four were delivered orally via encapsulated FMT;^41,49,50,64^ and two studies used a mixed methodology of endoscopic delivery or encapsulated FMT,^59^ or endoscopic route followed by encapsulated FMT.^51^ Twenty two studies used an aerobic preparation of FMT,^14,40,42,43,45–50,52,53,55–63^ one used an anaerobic preparation,^54^ and two did not specify.^41,51^ Two used a semi-anaerobic preparation of FMT (33, 37) in which feces was exposed to some oxygen during the procedure but attempts were made to minimize this; for example, prior to preparation, feces was stored in oxygen-depleted saline solution (36). Five studies used fresh feces, four used frozen feces, and two protocols allowed for use of fresh or frozen feces. One protocol did not describe whether feces were fresh or frozen.^63^

#### Dose

Dosing was inconsistently described. Twelve studies did not provide clear information regarding amount of stool used. Fourteen studies reported on the initial sample of fresh stool, whilst eight described the amount of “suspension” used, which consisted of filtered stool diluted with normal saline and sometimes mixed with a cryoprotectant such as glycerol. Doses of 12 g-250 g of fresh stool were reported in the 14 studies that did provide these data.

#### Adjunctive treatments

A wide range of adjunctive treatments were employed. Fourteen studies used bowel preparation,^14,39,42,43,45,47,52–56,58,60^ and six studies used antibiotics.^44,46,48,55,56,59,62^ Ren et al^63^ did not state whether bowel preparation was used and Herfarth et al^51^ did not report whether any adjunctive treatments were used.

#### Donor methods

Four studies used multiple donors (i.e. a pooled sample),^14,53,54,64^ two did not adequately describe whether single or multiple donors were used,^46,63^ and the remaining 20 studies used single donors. Nineteen studies used non-related donors, two used related donors only,^55,62^ one used either,^56^ and four did not specify.^46,57,63,64^

#### Screening protocol

Donor screening protocols overall were incompletely and poorly described. Where screening was stated as occurring, the methods for screening were frequently not provided. However, the more recent studies tended to have better reporting of screening protocols and more comprehensive screening. Fourteen studies specifically screened for metabolic risk factors, but only seven specifically described screening for mental illnesses.

### Study results

#### Efficacy

Results were categorized by disorder and are summarized in Table 2. Of the 26 included studies, 10 reported significant results for their primary outcome measures, where these related to clinical efficacy. These 10 studies related to functional gut disorders,^14,40,61,64^ Hepatitis B,^63^ IBD,^53,54,57^ antibiotic-resistant organisms,^46^ and metabolic syndrome.^52^ The evaluated conditions were highly heterogeneous, even within groups. Nonetheless, it was possible to perform meta-analyses for two groups of disorders: IBS, and active UC.

**Table 2.** Summary of primary outcomes of included studies

Author/date	Study details	Population details	Description of Intervention	Primary outcome measure (POM) relating to clinical efficacy (or relevant secondary outcome measures where POM did not relate to efficacy)	Results for outcome measures of clinical efficacy
Functional gut disorders					
Tian et al, 2017^40^	Study design: Randomized, single-blind controlled trial Country: China Sample size (n): N = 60 (FMT = 30, control = 30) Follow-up period: 12-weeks	Specific disorder: Slow transit constipation Age in years (mean): FMT group: 53.1 Control group: 55.4	Intervention: Frozen FMT delivered daily via NJT for 6 days plus TAU. Control: TAU	POM: Clinical cure rate (proportion of participants with an average of 3 or more complete spontaneous bowel movements per week during the 12-week follow-up).	Favors FMT The cure rate for the FMT group was 36.7% compared with 13.3% for the control group, (*P* = .04).
Johnsen et al, 2017^41^	Study design: Double blind, randomized placebo controlled parallel group, single center trial. 2:1 randomization Country: Norway Sample size (n): N = 83 (FMT = 45, control = 29) Follow-up period: 12-months	Specific disorder: IBS (excluded dominating constipation group) Age in years (median): FMT group: 43 Control group: 45	Intervention: bowel preparation and loperamide given prior to frozen FMT via colonoscopy. Control: Autologous FMT.	POM: Clinical response, defined as symptom relief of more than 75 points assessed by IBS-SSS, 3 months after FMT.	Favors FMT 36/55 (65%) participants in the FMT group compared with12/28 (43%) in the placebo showed a clinical response at 3 months (*p* = 0 · 049).
Halkjaer et al, 2018^45^	Study design: double blind placebo controlled trial for FMT capsules in IBS. 1:1 randomization Country: Denmark Sample size (n): n = 52 (FMT = 26, control = 26) Follow-up period: 6-months	Specific disorder: IBS Age in years (mean): FMT group: 37.28 Control group: 35.54	Intervention: 25 capsules of frozen encapsulated FMT daily for 12 days while fasting. Bowel preparation given the day prior to first treatment. Control: As above, but with placebo capsules used	POM: Reduction of IBS-SSS between baseline and 3-month follow up in the treatment group compared with the placebo group.	Favors placebo There was a significant difference in change in IBS-SSS groups between the FMT and placebo groups favoring the placebo group (*p* = .012).
Aroniadis et al, 2019	Study design: double blind, randomized, placebo controlled crossover trial Country: America Sample size (n): n = 48 (FMT = 25, control = 23) Follow-up period: 12–24 weeks	Specific disorder: IBS Age in years (mean): FMT group: 33 Control group: 42	Intervention: 25 capsules of FMT (daily) for 3 days plus TAU. Proton pump inhibitor prior to FMT. Control: Treatment as usual, plus placebo.	POM: Difference in IBS-SSS between the groups at 12 weeks	No significant result The difference in IBS-SSS and psychiatric outcome measures (HADS) scores between the FMT group and the control group were not significant.
El-Salhy et al, 2019	Study design: a double blind, randomized, placebo-controlled study Country: Norway Sample size (n): N = 165 (30 g FMT = 55, 60 g FMT = 55, control = 55) Follow-up period: 3 months	Specific disorder: IBS Age in years (mean): 30 g FMT group: 39.2 60 g FMT group: 39.3 Control group: 41.2	Intervention: TAU plus NGT FMT of 30 or 60 g. Control: As above, but 30 g autologous FMT used.	POM: Reduction in the IBS-SSS total score of ≥50 points at 3 months following transplantation	Favors FMT Responses occurredafter 3 months 30 g FMT and 60 g FMT groups (*p* < .0001). There was a significant improvement in the mental health sub-score of FAS in both groups at 3 months compared with placebo (*p* < .05).
Holster et al, 2019	Study design: a double blind, randomized, placebo-controlled study Country: Sweden Sample size (n): N = 16 (FMT = 8, control = 8) Follow-up period: 6 months	Specific disorder: IBS Age in years (median): FMT group: 34 Control group: 39	Intervention: Bowel preparation followed by colonoscopic FMT. Control: As above, but autologous FMT.	POM: Effect on IBS symptoms using the IBS version of the GSRS-IBS.	No significant result No significant differences in GSRS-IBS scores, anxiety or depression symptoms (HADS) between the allogenic and autologous groups were found.
Hepatic disorders					
Philips et al, 2018^14^	Study design: Retrospective cohort study Comments on study design: four arms of study and unclear allocation of groups Country: India Sample size (n): N = 51 (FMT = 16, steroids 8, nutritional support 17, pentoxifylline 10) Follow-up period: 90 days	Specific disorder: Severe alcoholic hepatitis Age in years (mean): FMT group: 47.6 Control groups: steroids 44.3, nutrition 49.6, pentoxifylline 48.7	Intervention: Fresh FMT daily for 7 days via NDT. Fasting before and after procedure. Control: Three control groups consisting of alternative treatments were used (steroids, nutrition or pentoxifylline therapy)	POM: Survival at 90 days.	No significant result There was no significant difference in 90-day survival between the groups.
Ren et al, 2017^46^	Study design: case controlled, single blind, open-label pilot trial Comments on study design: Data analyst blinded to study design, participants allocated into groups based on their treatment preferences (for FMT or not) Country: China Sample size (n): N = 18 (FMT = 5, control = 13) Follow-up period: 32–36 weeks	Specific disorder: Hepatitis B Age in years (median): FMT group: 27 Control group: 33	Intervention: FMT via NDT delivered every 4 weeks (for 1–7 treatments) plus TAU Control: TAU	POM: HbeAg clearance, defined as the loss of HbeAg.	Favors FMT 4/5 participants in FMT group achieved clearance of HbeAg, compared to 0/13 of the control group (*p* = .0002).
Bajaj et al, 2017	Study design: An open-label, randomized clinical trial Country: America Sample size (n): N = 20 (FMT = 10, control = 10) Follow-up period: 12–15 months	Specific disorder: Recurrent hepatic encephalopathy Age in years (mean): FMT group: 64.5 Control group: 62.9	Intervention: TAU, plus 5 days of pre-treatment with antibiotics (metronidazole, ciprofloxacin and amoxicillin), followed by a 12 hour washout, then FMT via retention enema. Control: TAU	POM did not relate to clinical efficacy. Relevant clinical outcomes included: changes in cognitive function at day 20, cirrhosis severity (MELD score, albumin) and changes in liver function	POM not relevant to clinical efficacy A significant improvement was observed in cognitive outcomes for FMT compared with control on both PHES total score (*p* = .003) and EncephalApp Stroop (*p* = .01). No significant changes were observed in cirrhosis severity or liver function for either group.
Bajaj et al, 2019	Study design: A Phase 1, Randomized, single-blind, Placebo-Controlled Trial Country: America Sample size (n): N = 20 (FMT = 10, control = 10) Follow-up period: 5 months	Specific disorder: Age in years (mean): FMT group: 63.3 Control group: 64.2	Intervention: 15 capsules of frozen, encapsulated FMT. Control: As above, but placebo capsules used, containing 80% cocoa butter, 20% glycerol and brown food coloring.	POM did not relate to clinical efficacy. Relevant clinical outcomes included: changes in cognitive testing on PHES and EncephalApp, serum LBP, and changes in duodenal mucosal expression of inflammatory cytokines, barrier proteins, and AMPs	POM not relevant to clinical efficacy Following intervention, a significant improvement in cognitive function was observed in FMT group, but not the control group compared to baseline for EncephalApp score (*p* = .02), but not PHES score. A significant post-treatment reduction in LBP was also observed in the FMT group (*P* < .009), but not the control group. No significant change in duodenal expression of inflammatory cytokines, barrier proteins or AMPs was observed.
Inflammatory Bowel Disease					
Ishikawa et al, 2017^39^	Study design: open-label, non-randomized prospective control study Country: Japan Sample size (n): N = 41 (FMT = 21, control = 20) Follow-up period: 4 weeks	Specific disorder: UC Age in years (mean): FMT group: 40.4 Control group: 44.7	Intervention: 2 weeks of antibiotics followed by fresh colonoscopic FMT (bowel preparation given prior), followed by scopolamine. Control: antibiotics only	POM: Clinical response (CAI of <10, and decrease of 3 or less) and clinical remission (CAI of 3 or less) at baseline compared with the four week follow up.	No significant result No significant difference was observed either in clinical response or clinical remission between the FMT and control groups.
Rossen et al, 2015^37^	Study design: Single center, randomized (1:1), double blind trial Country: The Netherlands Sample size (n): N = 50 (FMT = 25, control = 25) Follow-up period: 12 weeks	Specific disorder: Active UC Age in years (mean): Data not provided	Intervention: 2 doses fresh FMT via NDT 3 weeks apart, each preceded by bowel preparation. Control: As above, but autologous FMT used.	POM: Clinical remission at 12 weeks (SCCAI score < or = 2, and > or = 1 point improvement on the combined Mayo endoscopic score of sigmoid and rectum) compared to baseline.	No significant result There was no significant difference between the FMT group and control groups regarding clinical remission at 12 weeks.
Kump et al, 2017^42^	Study design: Open label, prospective, non-randomized controlled study Comments on study design: means of allocation not described Country: Austria Sample size (n): N = 27 (FMT = 17, control = 10) Follow-up period: 90 days	Specific disorder: Therapy-refractory, active UC Age in years (mean): FMT group: 44 Control group: 36	Intervention: Pre-treatment with antibiotics followed by 5 treatments of fresh FMT given 14 days apart. Bowel preparation given prior to first FMT only. First FMT delivered colonoscopically, and subsequent FMTs via sigmoidoscopy. Control: Antibiotics only.	POM: Mayo Score at the 90-day follow up point between the two groups, wherein a reduction in total Mayo score by three or more was considered a clinical response, and a score of two or less was considered remission	P-values/significance not described. 10/17 (59%) of the FMT group achieved a clinical response and four participants (24%) achieved clinical remission compared to 1/10 (10%) in the control group achieving partial response. *P*-values not provided, hence significance unclear.
Moayyedi et al, 2015^43^	Study design: double blind, placebo controlled, parallel design study (1:1) Country: Canada Sample size (n): N = 75 (FMT = 38, control = 37) Follow-up period: 12 months	Specific disorder: Active UC Age in years (mean): FMT group: 42.2 Control group: 35.8	Intervention: Fresh or frozen FMT given as retention enema weekly for 6 weeks. Control: As above, but water used as FMT placebo.	POM: UC remission (Mayo score <3 and endoscopic Mayo score 0) at week 7 compared with baseline.	Favors FMT At week 7, UC remission in the FMT group was 9/38 (24%) compared with 2/37 (5%) in the placebo group (*p* = .03).
Paramsothy et al, 2017^44^	Study design: multicentre, double-blind, randomized, placebo-controlled parallel design (1:1) Country: Australia Sample size (n): N = 81, FMT = 41, control = 40 Follow-up period: 8 weeks	Specific disorder: Active UC Age in years (median): FMT group: 35.6 Control group: 35.4	Intervention: Frozen FMT delivered via colonoscopy on day 1, followed by daily self-administered enemas of frozen FMT delivered 5 times per week for 8 weeks. Control: As above, but placebo colonoscopy and enema used (saline and glycerol).	POM: Steroid free clinical and endoscopic remission (total Mayo score ≤2, with all sub-scores ≤1, and ≥1 point reduction from baseline in endoscopy sub-score) at week 8 compared with baseline.	Favors FMT At week 8, remission rates in the FMT group were 27% compared with 8% in the placebo group, a RR of 3.6, (95% CI 1.1–11.9, *p* = .021).
Sokol et al, 2020	Study design: a multicentre, randomized, single-blind placebo-controlled trial Country: France Sample size (n): N = 17 (FMT = 8, control = 9) Follow-up period: 24 weeks	Specific disorder: Crohn’s disease Age in years (mean): FMT group: 31.5 Control group: 34	Intervention: Bowel preparation followed by colonoscopic FMT. Control: As above, but “physiological serum” used as placebo FMT.	POM did not relate to clinical efficacy. Relevant clinical outcomes included: clinical flare rate, change in CDEIS, CRP level, leukocyte level, or fecal calprotectin.	POM not relevant to clinical efficacy The CDEIS decreased significantly 6 weeks after FMT (*p* = .03) but not after sham. There was no significant difference in clinical fare rate, fecal calprotectin, leukocyte level or CRP level between groups.
Costello et al, 2019	Study design: a multi-center, double blind, randomized, controlled trial Country: Australia Sample size (n): N = 73 (FMT = 38, control = 35) Follow-up period: 12 months	Specific disorder: Active UC Age in years (median): FMT group: 38.5 Control group: 35	Intervention: 3 L polyethlene glycol bowel preparation given the night before, and 2 mg loperimide immediately prior. FMT consisted of frozen pooled donor stool via colonoscopy followed by 2 enemas on day 3 or 4 and one on day 6 or 7. Control: As above, but with autologous FMT	POM: Steroid-free remission at week 8 defined as 1. Total Mayo score of ≤ 2 AND 2. Mayo endoscopic score of ≤ 1	Favors FMT The primary outcome was achieved in 12/38 (32%) of the donor FMT group compared with 3/35 (9%) of autologous FMT group (*p* = .03).
Sood et al, 2019	Study design: a pilot double blind, randomized, placebo controlled study Country: India Sample size (n): N = 61 (FMT = 31, control = 30) Follow-up period: 48 weeks	Specific disorder: Inactive UC (maintenance) Age in years (mean): FMT group: 33 Control group: 34.6	Intervention: FMT delivered via colonoscopy every 8 weeks for 6 treatments plus TAU. Bowel preparation with polyethylene glycol lavage the night prior. Control: As above, but placebo colonoscopy given (saline with food dye).	POM: Maintenance of steroid-free clinical remission (Mayo score ≤2, all sub scores ≤ 1) at week 48. Relevant secondary clinical end points included: achievement of endoscopic remission (endoscopic Mayo score 0), histological remission (Nancy grade 0, 1) and change in inflammatory markers (ESR and CRP) at week 48.	No significant result for POM Nil significant difference in maintenance of steroid free clinical remission between groups. However, significant results were achieved for endoscopic remission (*p* = .026), histological remission (*p* = .033), and change in inflammatory markers (*p* < .001).
Antibiotic-resistant organisms					
Saidani et al, 2019	Study design: A matched case-control retrospective study (2 controls per case) Country: France Sample size (n): N = 30 (FMT = 10, control = 20) Follow-up period: 6 months	Specific disorder: CPE Age in years (mean): FMT group: 59.2 Control group: 60.3	Intervention: FMT via NGT, plus a nasopharangeal decolonization (8 days prior), bowel wash (5 days prior and 1 day prior), antibiotics for 5 days prior, and proton pump inhibitor (1 day prior and day of FMT). Control: TAU	POM: Delay in negativation of rectal-swab cultures.	Favors FMT At day 14 post-FMT, 8/10 treated patients (80%) achieved the POM, compared with 2/20 (10%) of the control group (*p* < .001) in the clearance rate between both groups.
Huttner et al, 2019	Study design: A multi-center (International) randomized, open-label, superiority trial Country: International (Swizerland, France, Israel, The Netherlands) Sample size (n): N = 39 (FMT = 22, control = 17) Follow-up period: 150–210 days	Specific disorder: CPE and ESBL Age in years (median): FMT group: 70 Control group: 64	Intervention: Colistin sulfate and neomycin sulfate tablets for 5 days followed by FMT (either capsules or NGT). Control: TAU	POM: Detectable intestinal carriage of ESBL/CPE by stool culture 35–48 days after randomization	No significant result for POM Nil significant difference between groups in intestinal ESBL or CPE rates following treatment.
Obesity or metabolic syndrome					
Kootte et al, 2017^36^	Study design: Double blind, randomized controlled trial of obese metabolic syndrome subjects Country: The Netherlands Sample size (n): N = 44 (FMT = 26, control = 12) Follow-up period: 18 weeks	Specific disorder: Metabolic Syndrome Age in years (median): FMT group: 54 Control group: 54	Intervention: Participants were fasted and received bowel preparation prior to fresh FMT via NDT. Control: As above, but participants received autologous FMT.	POM did not relate to clinical efficacy. Relevant clinical outcomes included: metabolic changes, insulin sensitivity and plasma metabolites at 6 and 18 weeks following FMT.	POM not related to clinical efficacy Significant improvement in insulin sensitivity was observed in FMT group at 6 weeks (*p* < .05), but not at 18 weeks.
Vrieze et al, 2012	Study design: a double blind, randomized controlled pilot study Country: The Netherlands Sample size (n): N = 18 (FMT = 9, control = 9) Follow-up period: 6 weeks	Specific disorder: metabolic syndrome (insulin sensitivity) Age in years (mean): FMT group: 47 Control group: 53	Intervention: Participants were fasted from the night before. Bowel lavage with polyethylene glycol solution given prior to FMT via NDT. Control: As above, but autologous FMT used.	POM: Change in insulin sensitivity at 6 weeks.	Favors FMT Peripheral insulin sensitivity improved at week 6 compared with baseline for the FMT group (*p* < .05), but not the control group. There was no significant change in hepatic insulin sensitivity at week 6, diet composition, resting energy expenditure, or counter-regulatory hormones.
Smits et al, 2019	Study design: a double blind, randomized controlled pilot study Country: The Netherlands Sample size (n): N = 20 (FMT = 10, control = 10) Follow-up period: 2 weeks	Specific disorder: metabolic syndrome (TMAO production) Age in years (mean): FMT group: 52.3 Control group: 57.7	Intervention: Bowel lavage, followed by FMT via NDT. Control: As above, but autologous FMT used.	POM: TMAO production (as a possible indicator for cardiovascular disease risk)	No significant change in POM At 2 weeks, there was no significant difference from baseline in fasting plasma TMAO, 24 hour urinary TMA excretion, 24 hour urinary TMAO excretion, plasma d3-carnitine appearance or 24 hour urinary d3-TMA excretion for FMT or control groups.
Allegretti et al, 2019	Study design: a double blind, randomized, placebo-controlled, pilot study Country: America Sample size (n): N = 22 (FMT = 11, control = 11) Follow-up period: 26 weeks	Specific disorder: obesity without metabolic syndrome Age in years (mean): FMT group: 44.5 Control group: 43.2	Intervention: Initial dose of 30 FMT capsules and a maintenance dose of 12 capsules at week 4 and week 8. Control: As above, but placebo capsules used.	POM did not relate to clinical efficacy. Relevant clinical outcomes included: Obesity related biomarkers such as change in weight or short-chain fatty acids, and change in area under the curve for GLP1 or leptin at week 12	POM not related to clinical efficacy There was a significant between group change in area under the curve for leptin, with a larger increase in the placebo group at week 12 compared with baseline (*p* = .001). There was no significant change in mean BMI, or area under the curve for GLP1, or short chain fatty levels at week 12 for either group.
Other disorders					
Vujkovic-Cvijin et al, 2017^30^	Study design: open label, non-randomized, prospective controlled study Comments on study design: Participants were selected for having low CD4 counts on ART (but excluded if they were too low) Country: America Sample size (n): N = 8(FMT = 6, control = 2) Follow-up period: 24 weeks	Specific disorder: HIV Age in years (median): FMT group: 61 Control group: 64	Intervention: Bowel preparation given prior to frozen colonoscopic FMT. Control: TAU	POM is not clearly stated. Relevant clinical outcomes included: HIV-associated markers of inflammatory activation (CD38, HLA-DR, CD8 + T-cells, plasma rations of kynurenine to tryptophan, IL-6, sCD14) over time (from baseline up to 24 week follow up).	No significant result Nil significant changes in any of the inflammatory markers between FMT and control groups was observed at any of the follow up points compared with baseline.
Herfarth et al, 2019	Study design: a prospective, placebo controlled, double blind randomized, controlled trial Country: America Sample size (n): n = 6 (FMT = 4, control = 2) Follow-up period: 16 weeks	Specific disorder: antibiotic-dependant pouchitis Age in years (mean): FMT group: 39.25 Control group: 33.5	Intervention: Two boluses of endoscopic FMT followed by daily dosing of 6 FMT capsules for 14 days. Control: As above, but placebo FMT used.	POM did not relate to clinical efficacy. Relevant clinical outcomes included: clinical remission (defined as an mPDAI < 4 and no need for antibiotics in weeks 4, 8, and 16) and change in fecal calprotectin level	POM not related to clinical efficacy All patients experienced relapse (ie. remission rate of zero for both groups). There was no significant change in fecal calprotectin levels as data were only available for 5 participants.

POM – Primary Outcome Measure, TAU – treatment as usual, UC- Ulcerative Colitis, ASD – Autism Spectrum Disorder, RCT – Randomized Controlled Trial, NDT – Nasoduodenal Tube, NJT – Nasojejunal Tube, NGT – Nasogastric Tube, FMT – Fecal Microbiota Transplant, ESR – Erythrocyte sedimentation rate, CRP – C-Reactive Protein, IBS – Irritable Bowel Syndrome, CDEIS-Crohn’s Disease Endoscopic Index of Severity, CPE – Carbapenemase-Producing Enterobacteriaceae, ESBL – Extended spectrum beta-lactamase, HIV – Human Immunodeficiency Virus, HADS – Hospital Anxiety and Depression Scale, FAS – Fatigue Assessment Scale, GSRS – Gastrointestinal Symptom Rating Scale, DSR – Daily Stool Records, SCCAI – Simple Clinical Colitis Activity Index, CAI – Clinical Activity Index, IBS-SSS – Irritable Bowel Syndrome Severity Scoring System, HbeAg – Hepatitis B-e Antigen, mPDAI – modified pouch activity

#### Inflammatory bowel disease

There were eight studies of IBD, six of which were of active UC, the remaining two being of Crohn’s Disease (CD),^45^ and maintenance of remission in UC.^60^ Sokol et al^45^ conducted a randomized, single-blind, controlled trial comparing colonoscopic FMT with placebo in 17 adults with CD. There was a significant decrease in CD symptoms in the FMT group compared with placebo (*p* = .03).^45^ Sood et al^60^ conducted a double-blind, randomized-controlled trial (RCT) of colonoscopic FMT compared with placebo as maintenance treatment for inactive UC. The study did not find a significant difference in the primary outcome measure (steroid-free clinical remission) between groups (*p* = .111); however, significant between-group differences were reported in endoscopic remission (*p* = .026), histological remission (0.033), and change in inflammatory markers (*p* < .001) favoring FMT.^60^

#### Meta-analysis for ulcerative colitis subgroup

Six studies reported on active UC, which was sufficient to perform a meta-analysis for clinical remission, clinical response, endoscopic remission, and endoscopic response. Outcome measures were heterogeneous, were collected at different time points (between 7 weeks and 90 days), and used differing definitions of clinical response/remission and endoscopic remission/response. Five of six used Mayo score, whilst one used Clinical Activity Index (CAI) score. Definitions and data for clinical remission and response are summarized in Supplementary Table 1, and endoscopic remission and response are summarized in Supplementary Table 2.

**Figure 2.**
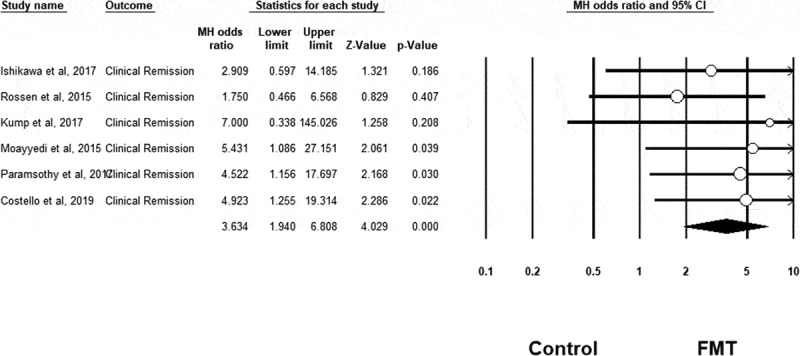
Clinical remission results

**Figure 3.**
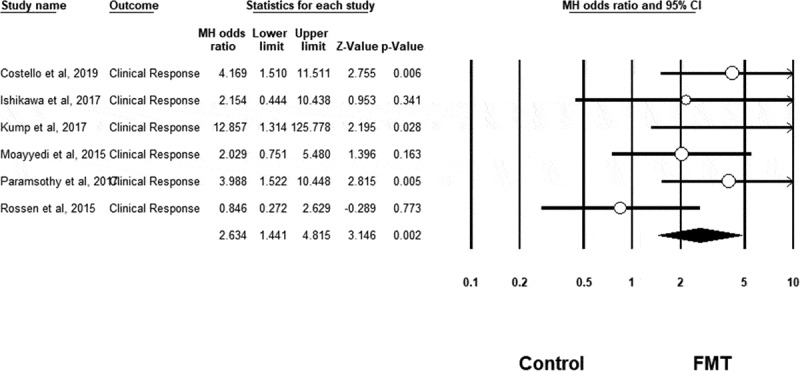
Clinical response results

**Figure 4.**
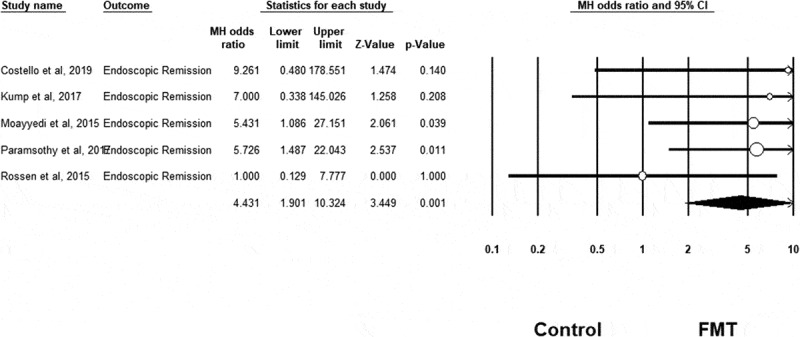
Endoscopic remission results

Meta-analysis confirmed that FMT was associated with a significant improvement in clinical remission rates in UC compared to control conditions (OR = 3.634, 95% CI = 1.940 to 6.808, n = 6 studies, I^2^=0%, *p* < .001) (see Figure 2). FMT was also associated with a significant improvement in clinical response rates in UC compared to control (OR = 2.634, 95% CI = 1.441 to 4.815, n = 6 studies, I^2^=33%, *p = *.002) (see Figure 3), as well as for endoscopic remission rates (OR = 4.431, 95% CI = 1.901 to 10.324, n = 5 studies, I^2^=0%, *p* = .001) (see Figure 4). However, FMT showed no significant improvement in endoscopic response rates in UC compared to controls (OR = 1.065, 95% CI = 0.432 to 2.625, n = 2 studies, I^2^=0%, *p* = .892) (see Supplementary Fig 1).

#### Functional gut disorders

In a trial of nasojejunally delivered FMT given daily for six days in adjunct to treatment as usual (TAU) for slow-transit constipation, Tian et al^61^ reported a clinical remission rate of 36.7% for the FMT group compared with 13.3% for the TAU control group (*p* = .04).

Five studies reported on IBS, which was sufficient to perform a meta-analysis for clinical response and average change in IBS-SSS. Different studies used different definitions of clinical response: four used IBS-SSS, and one used GSRS-IBS, mostly at 3 months. Definitions and data for clinical response and change in IBS-SSS are summarized in Supplementary Table 3.

Meta-analysis revealed no significant difference in IBS-SSS (Hedge’s g = 0.282, 95% CI = −1.373 to 1.937, n = 3 studies, I^2^=97%, *p* = .739) or clinical response (OR = 1.699, 95% CI = 0.273 to 10.588, n = 5 studies, I^2^=92%, *p* = .739) following FMT compared to control (see Supplementary material; Figures 2 and 3)

#### Psychiatric outcomes

Only three studies assessed psychiatric outcomes and all three were conducted in IBS populations. Two of the studies used the Hospital Anxiety and Depression Scale (HADS) to measure depression and anxiety symptoms and neither reported a significant change in symptoms between groups post-intervention. The third study used the mental health subscale of the Fatigue Assessment Scale (FAS), and reported a significant difference between the means of the group who received 30 g FMT (13.3, s.d. 3.1) compared with the placebo group (14.7, s.d. 3.4) at 1 month (*p* < .05), and the group who received 60 g FMT (13.1, s.d. 3.1), compared with the placebo group (14.5, s.d. 2.7) at 3 months (*p* < .05) in favor of FMT.

#### Hepatic disorders

Of the four studies in hepatic disorders, three had significant results for clinical efficacy favoring FMT over control, whilst the fourth did not report significant outcomes. In a trial of nasoduodenally delivered FMT every 4 weeks (for 1–7 treatments) plus TAU for Hepatitis B, Ren et al^63^ reported that four of the five participants achieved clearance of HbeAg, whereas all 13 of the TAU controls continued to have a positive HbeAg titer (*p* = .0002). Bajaj et al^48^ conducted an open-label RCT investigating FMT for recurrent hepatic encephalopathy using a retention enema compared with TAU and reported a significant improvement in two measures of cognitive outcomes in favor of FMT (*p* < .01 for both). Similarly, in a single-blind RCT of encapsulated FMT compared with placebo capsules, Bajaj et al^49^ found a significant improvement in cognitive outcomes for the FMT group but not the placebo group (*p* = .02). Philips et al^62^ conducted a retrospective cohort study comparing FMT with three control groups (steroids, nutritional support or pentoxifylline) for the treatment of severe alcoholic hepatitis, but found no significant improvement in the primary outcome of 90-day survival (*p* = .179).

#### Metabolic syndrome or obesity without metabolic syndrome

Four studies evaluated FMT for the treatment of metabolic syndrome or obesity without metabolic syndrome, and only one of these had significant results for clinical efficacy regarding the primary outcome. The remaining three showed significant results for secondary outcome measures, all in favor of FMT. Vrieze et al^52^ conducted a double-blind pilot RCT of nasoduodenally delivered FMT compared with autologous FMT for metabolic syndrome and reported a significant improvement in week 6 peripheral insulin sensitivity (*p* < .05) in favor of FMT, but not in hepatic insulin sensitivity (*p* = .08), diet composition, resting energy expenditure, or counter-regulatory hormones. In a double-blinded RCT examining nasoduodenally delivered FMT for metabolic syndrome, Kootte et al^39^ did not find significant differences in their primary outcome measure (change in intestinal microbiota in relation to insulin sensitivity at 18 weeks), nor did they observe a significant change in BMI or SCFA levels at any study time point. In terms of secondary outcomes, change in fecal microbiota composition at 6 weeks associated with improved peripheral insulin sensitivity (from 25.8 [19.3–34.7] to 28.8 [21.4–36.9] mmol kg/1 min/1, *p* < .05) in the allogenic FMT group, whereas autologous FMT had no effect (from 22.5 [16.9–30.2] to 20.8 [17.6–29.5] mmol kg/1 min/1, *p* > .5).

Smits et al^43^ conducted a double-blind pilot RCT of nasoduodenally delivered FMT compared with autologous FMT for TMAO production in participants with metabolic syndrome and did not find a significant difference between groups. In a double-blind pilot RCT of encapsulated FMT compared with placebo capsules for obesity-related biomarkers in participants with obesity but without metabolic syndrome, Allegretti et al found a significant between-group difference in area under the curve at week 12 for leptin compared with baseline (*p* = .001), but no significant change for other biomarkers of obesity.^50^

#### Antibiotic-resistant organisms

Two studies evaluated FMT for the treatment of colonization of antibiotic-resistant organisms, one demonstrating significant clinical efficacy of FMT over control and the other without significant outcomes. In a retrospective matched case-control study of nasogastric FMT compared with TAU for Carbapenemase-Producing Enterobacteriaceae (CPE), Saidani et al^46^ reported a significant delay in negativation of rectal swab cultures 2-weeks post-FMT compared with TAU (*p* < .001). Huttner et al^59^ conducted a multicentre, randomized, open-label, superiority trial of nasogastric or encapsulated FMT (treatment was site dependant), compared with TAU for CPE and Extended spectrum beta-lactamase (ESBL), but did not identify a significant between-group difference in the primary outcome measure for clinical efficacy (*p*-value not provided).

#### Other conditions

Two studies were not able to be grouped with the others. They evaluated FMT for the treatment of individuals with HIV and antibiotic-dependant pouchitis, respectively. Neither showed clinical efficacy.

### Safety data

There were variable quality and completeness of reporting of safety data for both serious adverse events (SAEs) and mild to moderate AEs, across studies (see Table 3). Studies had a follow up period ranging from four weeks^55^ to 1 year .^14^ **SAEs**

**Table 3.** Completeness of reporting of AE

Completeness of reporting of AE	Number reported (total studies, n = 26)
**Mild-moderate AE**	
Detailed reporting of AE	9 (34.6%)
Generic statement only or limited reporting of AE	9 (34.6%)
Not reported at all	8 (30.8%)
**SAE**	
Clearly described	23 (88.5%)
Not reported at all or not clearly reported	3 (11.5%)

Of the 26 included studies, 23 provided clear descriptions of SAEs. A total of 69 SAEs were reported from 12 studies; 26 occurred in participants allocated to receive FMT, and 43 in participants in the control groups (see Supplementary Table 4). Of the 26 SAEs that occurred in participants allocated to receive FMT, all but one was deemed unlikely to be related to the intervention. Twenty of these SAEs occurred in participants who received FMT via colonoscopy or enema, and six in those receiving FMT endoscopically or via capsules. When broken down by specific disorder, 17 of these SAEs occurred in participants with inflammatory bowel disease, three in participants with hepatic encephalopathy, five in participants with antibiotic resistant organisms and one in a participant with IBS.

### Mild to moderate AEs

Due to the inconsistent quality and completeness of reporting of mild to moderate AE, it was only possible to pool/summarize data for a small number of included studies (see Supplementary Table 5). These studies were related to IBS, (n = 4), UC (n = 2), slow transit constipation (n = 1), hepatic encephalopathy (n = 1), and metabolic syndrome (n = 1). As such a cross-indication assessment of adverse events was not possible as the data were insufficiently reported across disorders.

Similar rates of mild to moderate AEs were observed in participants allocated to FMT compared to the control groups (see Supplementary Table 5). However, the following AEs were more common in participants receiving allogenic FMT compared with those allocated to control groups: nausea (reported in 80% of FMT recipients compared with 72% in control groups), constipation (reported in 17.4% of FMT recipients compared with 2.4% in control groups), diarrhea (reported in 16.8% of FMT recipients compared with 6.7% in control groups), transient, or low-grade fever (reported in 8.4% of FMT recipients compared with 3.0% in control groups) and vomiting (reported in 5.9% of FMT recipients compared with 2.9% in control groups).

Incomplete reporting precluded comparison of AE rates between different routes of FMT; however, encapsulated FMT appears to have been the best-tolerated route.

#### Successful microbial “engraftment”

Microbiome analysis pre- and post-FMT was performed in 23 of the 26 included studies. All microbiome analyses used 16s RNA sequencing. The data relating to “engraftment” are summarized in Table 4. All 23 of 23 studies which measured microbiome analysis reported change in microbiome following FMT. Fourteen of the 23 studies reported whether the change in microbiota was toward the donor and, of these, 11 confirmed that the recipient microbiome did move toward the donor microbiome. The remaining three studies did not report significant results.

**Table 4.** Summary of “engraftment” of FMT

Study	Does the microbiome change in recipients following FMT?	Were the changes toward the donor microbiome?	Duration of microbiome changes	Was an association observed between microbiome changes and clinical outcomes?
Holster et al, 2019^52^	Yes	Yes	Changes appeared to persist for 8 weeks (final data point).	Yes
El Salhy et al, 2019^49^	Yes	Not described	Appeared changed at one month (only data point).	Yes
Aroniadis et al, 2019^34^	Yes	Yes	Changes appeared to persist for 12 weeks (final data point).	No
Costello et al, 2019^48^	Yes	Yes	Changes appeared to persist for 8 weeks, reduced by 12 months.	Yes
Sood et al, 2019^54^	Not measured	N/A	N/A	N/A
Herfarth et al, 2019^35^	Yes	Only in one of six recipients	Not clearly described.	Yes
Allegretti et al, 2019^65^	Yes	Yes	Changes appeared to persist for 12 weeks (final data point).	Not described
Vrieze et al, 2012^47^	Yes	Not described	Appeared changed at six weeks (only data point).	Yes
Smits et al, 2018^38^	Yes	Yes, “in some but not all participants” – further detail not provided.	Appeared changed at two weeks (only data point).	Not described
Bajaj et al, 2017^32^ and long term data reported in Bajaj et al, 2019^56^	Yes	Yes	Changes appeared to persist for over one year (reported in long term paper).	Not described
Bajaj et al, 2019^33^	Yes	Not measured	Appeared changed at day 30 (only data point).	Not described
Sokol et al, 2020^51^	Not significant overall. However when data from 2 participants was removed, a significant change was observed in the remaining 8 participants	Not significant	Not significant overall, however when data was corrected for 2 participants who were considered “treatment failures”, duration of changes were significant at 6 weeks for the FMT group, but microbiota were considered back to baseline at week 14.	Yes
Vujkovic-Cvijin et al, 2017^30^	Yes	Yes	Changes in microbiota were most significant between 2–4 weeks, and less significant by week 8.	Not described
Ren et al, 2017^46^	Yes	Not described	Not described.	Not described
Philips et al, 2018^14^	Yes	Not described	Changes appeared to persist for up to 90 days (study duration).	Not described
Kump et al, 2017^42^	Yes	Yes	Changes appeared to persist for 90 days (final data point).	No
Ishikawa et al, 2017^39^	Yes	Not described	Appeared changed at 4 weeks, but not measured beyond that.	Yes
Tian et al, 2017^40^	Not measured	N/A	N/A	N/A
Kootte et al, 2017^36^	Yes	Not described	Changes demonstrated at 6 weeks. No changes apparent at 18 weeks.	Yes
Paramsothy et al, 2017^44^	Yes	Yes	Changes persisted for 8 weeks after intervention finished (final data point).	Yes
Johnsen et al, 2017^41^	Not measured	N/A	N/A	N/A
Halkjaer et al, 2018^45^	Yes	Yes	3 months (final data point).	Not significant
Rossen et al, 2015^37^	Yes	Yes, but only in responders	Changes were observed at 12 weeks (final data point).	Yes
Moayyedi et al, 2015^43^	Yes	Not significant	Changes were observed at 6 weeks (only data point).	Not significant

Reporting on the extent or significance of microbiota changes was inconsistent across studies and the complexity of microbiome data analysis has meant it was not possible to answer the question of the extent to which the recipient microbiome changed toward the donor, as no clear quantification was provided by the included studies. As such, these data are not reported in Table 4.

Regarding longevity of the observed changes in recipient microbiota, it was not possible to answer this question in this review, as included studies either did not follow-up recipients for long enough, or did not measure microbiota changes frequently enough to be able to state the duration for which any changes were observed. However, with these limitations in mind, it appears that the demonstrated microbiome changes were transient and appeared to last between 2 weeks to 1 year following the intervention.

#### Correlation of “engraftment” with clinical findings

Fourteen of 23 studies reported on associations between “engraftment” and clinical outcomes, and of these, 12 studies had statistically significant results with 10 reporting a significant association between successful engraftment and clinical efficacy and two reporting no association between efficacy and engraftment. These data are summarized in Table 4.

### Risk of bias assessment

According to the Cochrane Risk of Bias tool^36^ (see Supplementary Table 6), nine studies were evaluated as “low risk,”^14,40–42,49,53,54,58,60,64^ six as “some concerns,”^39,42,43,45,50,52,57^ and five as “high risk.”^48,57–59,61^ Studies rated “high risk” were: Tian et al,^61^ due to incomplete reporting across most domains and inadequate randomization processes; Moayyedi et al^57^ due to likely inadequacy of blinding of participants as water enemas were used as placebo, which would likely be easily differentiated from true FMT by recipients; Bajaj et al^48^ and Huttner et al^59^ as the studies were open label, with an absence of blinding; and Herfarth et al,^51^ due to an absence of a statistical pre-analysis plan, and the fact that the trial was ceased after only six participants were randomized.

## Discussion

### Statement of principal findings

This review identified FMT trials for conditions other than CDI, with promising, albeit mixed, outcomes regarding efficacy and safety. Meta-analysis of UC studies found FMT to be superior to control conditions for active disease in terms of endoscopic remission, clinical remission, and clinical response. In contrast, meta-analysis of the five IBS studies did not yield significant results regarding symptoms or clinical response. Regarding clinical efficacy in other applications of FMT, studies were too heterogeneous to perform meta-analyses, but four yielded evidence of clinical efficacy in slow-transit constipation, Hepatitis B, colonization of CPE, and insulin sensitivity in metabolic syndrome. The impact of FMT on psychiatric outcomes was assessed in three studies of IBS patients, with one of these finding significant improvements.

This review also found that FMT was safe and well tolerated. Similar rates of mild to moderate AEs were observed in participants who received FMT compared to those allocated to control groups, while SAEs were more commonly reported in participants allocated to control/placebo groups.

Not all studies assessed or reported whether FMT results in successful engraftment of the donor microbiome into the recipient, but a majority of those that did report it confirmed a move toward the donor microbiome following FMT and that these changes persisted for up to 1 year. Furthermore, four of the five studies that reported on association between microbiome changes and clinical efficacy, four of five confirmed such an association. This suggests that FMT alters the recipient microbiome, and that it is possible that this change is a contributing factor to clinical efficacy.

### Strengths and weaknesses of the review

This review is the first systematic review to evaluate both safety and efficacy of FMT for all disorders other than CDI. This review aimed to recruit higher quality studies by excluding uncontrolled studies, which represent a majority of studies in this field. Whilst other reviews have been conducted with respect to safety of FMT for indications other than CDI, these reviews are either not recent,^65^ or were restricted to a single indication such as IBS^29–31^ and IBD.^32–34^ With respect to efficacy, whilst other reviews have been published for single indications, such as IBS^29–31^ and IBD,^32–34^ there have been no holistic reviews looking at all indications other than CDI. As far as the authors of this review are aware, this review also represents the most up to date systematic review and meta-analysis of the safety and efficacy of FMT for IBS.

However, the 26 studies included were heterogeneous and of mixed quality, with several using open-label designs and small samples. Encouragingly, more recently published studies appear to be of higher quality, using more robust study designs (such as double-blinded RCTs), and with clearer and more complete descriptions of study methodology.

It was possible to conduct meta-analyses for both IBS and active UC. However, due to the lack of consensus regarding outcome measures and small number of included studies, the results of these meta-analyses should be considered preliminary at this stage. Further, due to the low numbers, tests for publication bias (e.g. eggers regression and funnel plots) were unable to be carried out.

We were unable to undertake a quantitative analysis on the level of engraftment, given the gaps in data in the included studies. Future studies should evaluate microbial engraftment as a result of FMT, allowing for a systematic assessment.

This paper evaluated safety data across a range of indications, finding broadly that FMT is well tolerated and safe. However, due to the poor quality and incompleteness of reporting in several papers, a cross-indication analysis of safety data was not possible. We recommend future FMT studies report more clearly on mild to moderate and SAE.

With respect to SAE, these were observed more frequently in control group participants than those allocated to receive FMT, and of the SAE observed in FMT recipients, most considered to be unrelated to FMT. In understanding this finding, it should be noted that most SAE were likely due to the underlying disease process rather than the FMT procedure, a majority of reported SAE were flares of the disease in inflammatory bowel disease participants. Thus, FMT may have prevented disease flares.

### Implications for clinicians and policymakers

With respect to active UC, our meta-analysis revealed that FMT appears to be clinically efficacious compared to control conditions. Four of the six included studies used a gold-standard double-blind placebo-controlled RCT design, and all six included studies favored FMT over control conditions regarding clinical efficacy, notwithstanding limitations described above. Thus, the quality and consistency of outcomes appear to favor FMT in the treatment of active UC, making this is a promising area for research attention.

Evidence supporting the application of FMT in the treatment of IBS is more equivocal. Five studies included in this review showed mixed outcomes, with three reporting that FMT was favorable, and two finding that control conditions were more effective than FMT. Possible reasons for these mixed findings are discussed in depth in other review papers,^29–31^ which note, inter alia, that route of delivery, choice of placebo (i.e. inert vs autologous FMT), and patient group may have contributed.^29–31^ Suffice to say, there is no strong evidence at this stage that FMT is efficacious for the treatment of functional gut disorders.

This review also identified two additional studies relating to inflammatory bowel disease yielding significant outcomes regarding clinical efficacy, and four studies that evaluated FMT for metabolic syndrome or obesity that focused on biological outcomes rather than clinical efficacy. As such, these are identified as conditions of interest for further research only.

Regarding safety, FMT appears generally to be a safe and well-tolerated treatment, with orally administered FMT appearing to be the best-tolerated route. However, it is also important to note that on 13^th^ June, 2019 the American-based Food and Drug Administration (FDA) released a statement warning of the risks of FMT. They reported two cases (both immunocompromised patients) in whom antibiotic-resistant organisms, (specifically, Extended Spectrum Beta Lactamase-producing *Escherichia coli (E.coli))* were transferred via FMT, resulting in one death. In these cases, donor feces were not screened for antibiotic-resistant organisms.^66^ A further warning was issued on 12^th^ March 2020 advising of six cases of additional transmission of antibiotic-resistant organisms via FMT provided by a US-based stool bank (enteropathogenic Escherichia coli in two cases and Shigatoxin-producing Escherichia coli in four cases) and two deaths that occurred in recipients of FMT, but in which FMT may not have been the cause of death.^67^ It is now standard across widely accepted protocols in the United Kingdom, United States, and Australasia to screen thoroughly for antibiotic-resistant organisms. These recent serious incidents highlight the importance of adhering to rigorous screening protocols, such as the Openbiome Protocol in the US,^68^ the British Guidelines for donor screening,^69^ or the Australasian guidelines.^70^

### Unanswered questions, challenges for the field of FMT research, and future directions

Of all the uses of FMT for conditions other than CDI, the most promising at this stage is for active flares of UC. Further large scale, high-quality studies, utilizing consistent data points to measure primary outcomes, are urgently called for. Other indications with some promise include metabolic syndrome/obesity, antibiotic-resistant organisms, and certain hepatic disorders. Whilst published outside of the time range of the search performed for this review, a recent Phase I study investigating FMT for Alcohol Use Disorder showed safety and efficacy.^71^ Thus, we watch with interest the growing field of FMT for hepatic disorders.

However, on the whole, studies in these emerging areas are heterogeneous and generally of poor quality, with most using open-label designs and only one study using clinical efficacy as a primary outcome measure. Again, further high-quality research, using larger sample sizes and double-blinded, placebo-controlled designs, and that use clinical efficacy endpoints as a primary outcome, are needed for these emerging indications. Furthermore, no studies evaluated the use of FMT for psychiatric conditions, an area of great importance given growing interest and data supporting the relationship between mental health and the gut microbiome (“the microbiota-gut-brain axis”).^7,72^ Much also remains unknown about the ideal methodological design for studies of FMT for conditions other than *Clostridium difficile* infection. For one, an important question remains around choice of placebo. Nine studies selected an inert placebo, such as water mixed with glycerol and food dye, whilst eight opted for autologous FMT as a placebo. There is some evidence to suggest that even autologous FMT may have an impact on gut microbiota,^58^ which may confound results. This needs to be further explored but presents an argument against using autologous FMT as a placebo in future studies aimed at determining efficacy of FMT compared with an inactive control. Follow-up periods to assess long-term safety, engraftment, and metagenomics are also an important consideration for study design. The authors of this review suggest a follow up period of at least 6 months to adequately monitor for safety and long-term AE.

## Conclusions

This systematic review and meta-analysis provide preliminary data that FMT may be safe and effective for several conditions other than CDI. Preliminary meta-analyses suggest efficacy for outcomes related to active ulcerative colitis but not IBS. Hepatic disorders, metabolic syndrome/obesity, and antibiotic-resistant organisms were also identified as emerging areas of interest for FMT research. Regarding safety, there was little difference in SAEs between participants allocated to receive FMT and those allocated to control groups. With respect to mild to moderate adverse events, similar rates were also observed in treatment and control groups. These encouraging pilot outcomes provide preliminary support for further high-quality research in these areas.

## Supplementary Material

Supplemental Material
