# Supplementary material for: Efficacy and safety of fecal microbiota transplantation for the treatment of diseases other than Clostridium difficile infection: a systematic review and meta-analysis
Source: Gut Microbes. 2020 Dec 19;12(1):1854640. doi: 10.1080/19490976.2020.1854640 (PMC7757860; doi:10.1080/19490976.2020.1854640)
Supplement: Supplemental Material [file KGMI_A_1854640_SM0716.docx]

**Supplementary Table 1– Clinical remission and clinical response data**

|  |  | Intervention group | | | Control group | | |
| --- | --- | --- | --- | --- | --- | --- | --- |
|  | Clinical response/remission definition | Total sample size (n) | Clinical response (n) | Clinical remission | Total sample size (n) | Clinical response | Clinical remission |
| Ishikawa et al, 2017 ([47](#_ENREF_47)) | clinical response: CAI of <10, and decrease of 3 or less, clinical remission: CAI of 3 or less | 17 | 14 | 6 | 19 | 13 | 3 |
| Rossen et al, 2015 ([38](#_ENREF_38)) | clinical remission at 12 weeks: SCCAI score < or = 2, and > or =1 point improvement on the combined Mayo endoscopic score of sigmoid and rectum compared to baseline  Clinical response: reduction of > or =1.5 points on SCCAI at 12 weeks compared with baseline | 23 | 11 | 7 | 25 | 13 | 5 |
| Kump et al, 2017 ([48](#_ENREF_48)) | A reduction of the total Mayo score by ≥3 points was considered as a clinical response, whereas a drop of the Mayo score to ≤2 points was considered as remission. Participants with a response but no remission are denoted as partial responders. | 17 | 10 | 4 | 10 | 1 | 0 |
| Moayyedi et al, 2015 ([49](#_ENREF_49)) | Remission: at week 7 Deﬁned as full Mayo Clinic score <3 and an endoscopic Mayo Clinic score = 0. Response: At week 7, Deﬁned as a reduction in full Mayo clinic score of at least 3 points. | 38 | 15 | 9 | 37 | 9 | 2 |
| Paramsothy et al, 2017([41](#_ENREF_41)) | “steroid free remission and endoscopic remission/response”: Total Mayo score ≤2, with all sub-scores ≤1, and ≥1 point reduction from baseline in endoscopy sub-score. “Steroid-free response”: Decrease of ≥3 points or ≥50% reduction from baseline (or both) in combined Mayo sub-scores for rectal bleeding plus stool frequency. | 41 | 22 | 11 | 40 | 9 | 3 |
| Costello et al, 2019 ([42](#_ENREF_42)) | Steroid free remission: at week 8 defined as Total Mayo score of ≤ 2 Mayo endoscopic score of ≤ 1  Clinical response: ≥3 point reduction in total Mayo score at week 8 and 1 year | 38 | 21 | 12 | 35 | 8 | 3 |

**Supplementary Table 2 – Endoscopic remission and endoscopic response data**

|  | Endoscopic response/remission definition | Intervention group | | | Control group | | |
| --- | --- | --- | --- | --- | --- | --- | --- |
|  | Endoscopic remission = Endoscopic Mayo score of 0 or 1 ([57](#_ENREF_57)) | N= | Endoscopic response | Endoscopic remission | N= | Endoscopic response | Endoscopic remission |
| Ishikawa et al, 2017 ([47](#_ENREF_47)) |  | 21 | DNP | DNP | 20 | DNP | DNP |
| Rossen et al, 2015 ([38](#_ENREF_38)) | Response: In all nine participants the last observation carried forward criteria were applied to calculate the 12-week endoscopic response rate  Remission: Endoscopic Mayo score =0 at week 12 | 23 | 8 | 2 | 25 | 9 | 2 |
| Kump et al, 2017 ([48](#_ENREF_48)) | Remission: Endoscopic Mayo score of 0 or 1 at 90 days | 17 | DNP | 4 | 10 | DNP | 0 |
| Moayyedi et al, 2015 ([49](#_ENREF_49)) | Remission: Endoscopic Mayo score of 0 at week 7 | 38 | DNP | 9 | 37 | DNP | 2 |
| Paramsothy et al, 2017([41](#_ENREF_41)) | Response: Mayo endoscopy sub-score ≤1, with ≥1 point reduction from baseline.  Remission: Endoscopic Mayo score = 0 at week 8 | 41 | 5 | 13 | 40 | 4 | 3 |
| Costello et al, 2019([42](#_ENREF_42)) | Remission: Endoscopic Mayo score < 1 at week 8 | 38 | DNP | 4 | 35 | DNP | 0 |

DNP – data not provided

**Supplementary Fig 1 – Endoscopic response results**

**
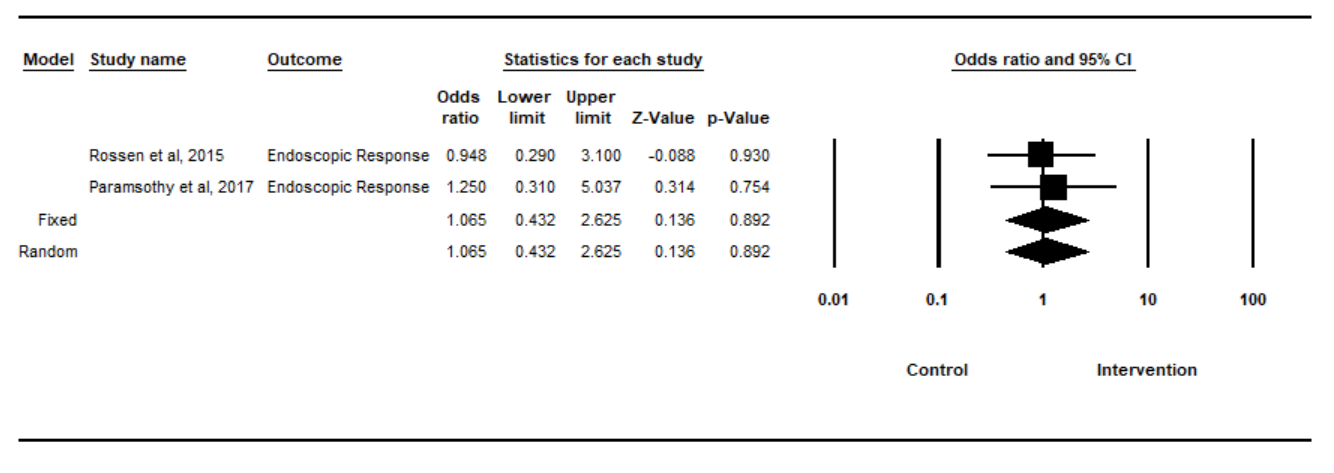
**

**Supplementary Table 3 – Clinical remission and clinical response data**

|  | Study population | Clinical response definition | Intervention group | | | Control group | | |
| --- | --- | --- | --- | --- | --- | --- | --- | --- |
|  |  |  | N= | Average change in IBS-SSS | Clinical response | N= | Change in IBS-SSS | Clinical response |
| Halkjaer | Mod-severe IBS (IBS-SSS > or =175) | Reduction in IBS-SSS of more than 50 at 3 months | 22 | Mean: -52.45, s.d. 97.72, p=0.012 | Reduction by >50: 8/22 (36.4%), p=0.008 | 24 | Change in IBS-SSS:  -125.71, s.d. 90.85, p=0.012 | Reduction by >50: 19/24 (79.2%), p=0.008 |
| Johnsen | Mod-severe IBS (IBS-SSS > or =175) with diarrhoea or diarrhoea and constipation (dominating constipation excluded) defined by ROME III criteria | Symptom relief of more than 75 in IBS-SSS at 3 months | 55 | DNA | 36/55 (65%), p=0.049 | 28 | DNA | 12/22 (43%), p=0.049 |
| Aroniadis | Mod-severe IBS-D (IBS-SSS>175) | Reduction of IBS-SSS of more than 50 at 12 weeks | 25 | Baseline (mean): 282, SD 65  12 weeks (mean): 221, SD 105, p=0・65  Difference: 61 | 11/22 (50%), p=0.46 | 23 | Baseline (mean): 309, sd 64  12 weeks (mean): 236, sd 95; p=0・65  Difference:73 | 14/23 (61%), p=0.46 |
| El-Salhy | Mod-Severe IBS (175 or more on IBS-SSS) Rome IV criteria for IBS | Decrease of 50 points or more on IBS-SSS at 3 months | 30g FMT – 54  60g FMT - 55 | **30g FMT (**Mean):  At baseline: 311.8, sd 76.8  At 3 months: 186.3, sd 109.0, p<0.001  Difference: 125.5  **60g FMT** (Mean):  At baseline:313.3, sd 87.3  At 3 months: -166.8, sd 117.9, p<0.001  Difference: 146.5 | 30g: (41/54) 76.9%, p<0001  60g: (49/55) 89.1%, P<0.0001 | 55 | Baseline (mean): 315.2, sd 77.1  3 months: 307.0, sd 87.1, p<0.001  Difference: 8.2 | 13/55 (23.6%) |
| Holster | Rome III criteria for IBS | A decrease of at least 30% in the  total GSRS-IBS symptom score after FMT | 8 | median: 4wks: -70.6 (IQR -90.1to -10.5), p<0.05  8wks:-69.3 (IQR-77.2 to -19.3), p<0.05 | 4/8 (50%), p=0.282 | 8 | DNA | 1/8 (12.5%), p=0.282 |

DNA – Data not available

**Supplementary Fig 2 – Difference in IBS-SSS results**


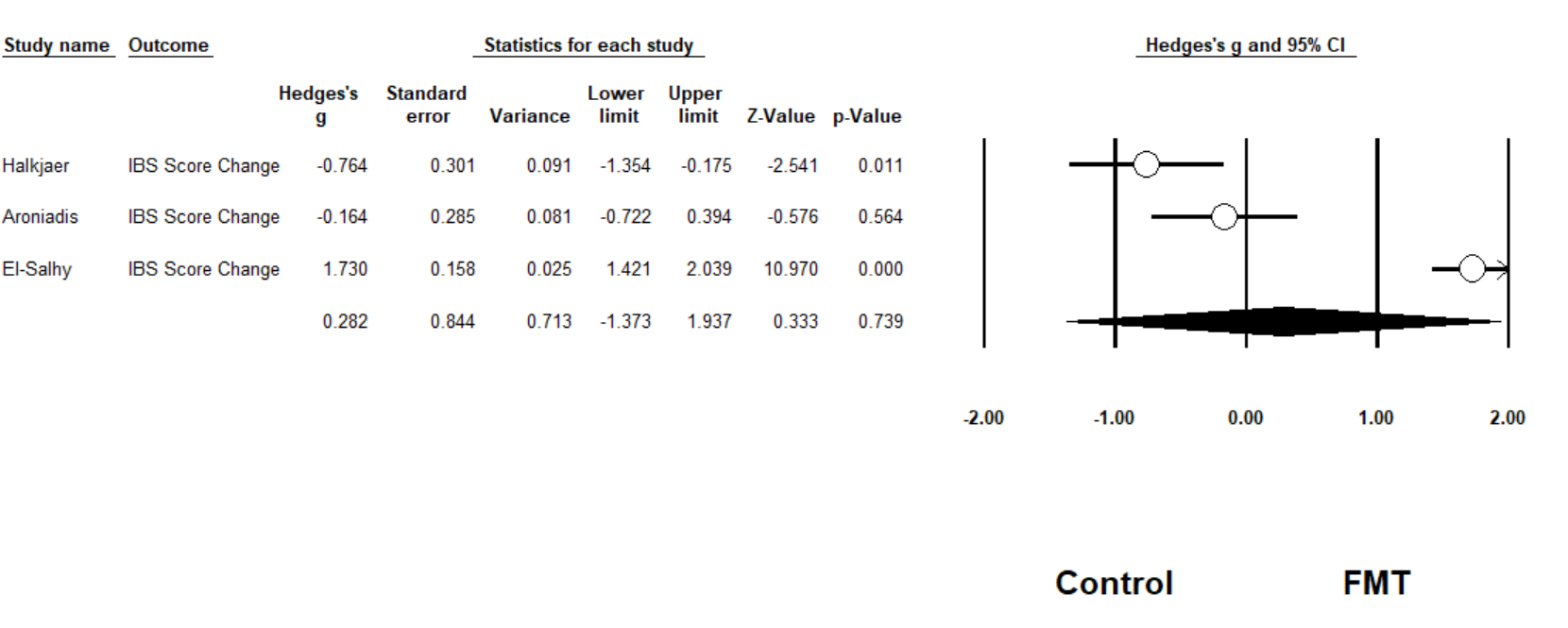


**Supplementary Fig 3 – Clinical response results**


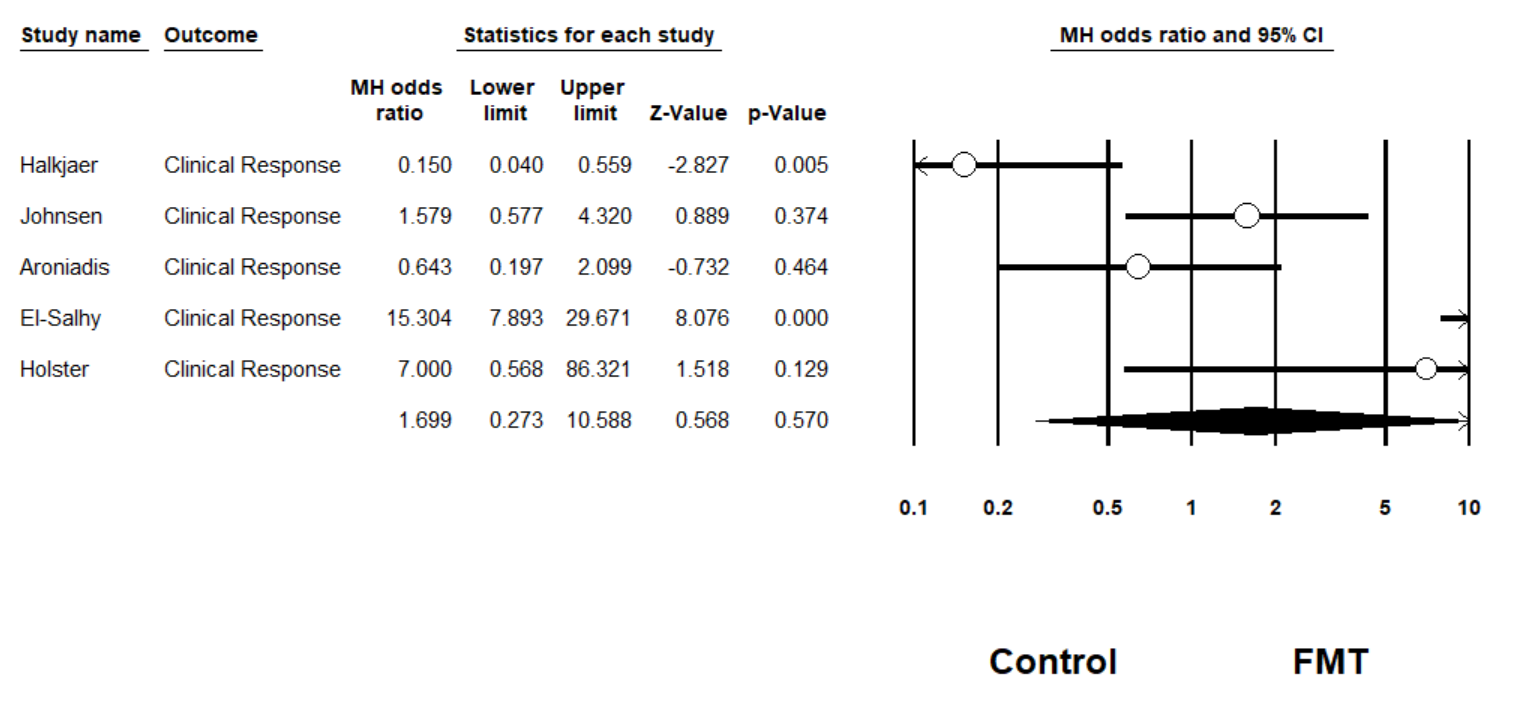


**Supplementary Table 4 – SAE**

|  |  | SAE (n) | | Likelihood of relationship of AE to FMT | | |
| --- | --- | --- | --- | --- | --- | --- |
| Study | SAE type | FMT group | Control group | Unlikely | Possible | Probable |
| Johnsen et al, 2017 ([44](#_ENREF_44)) | Self-limiting nausea and vertigo | 1 | 0 | X |  |  |
| Rossen et al, 2015 ([38](#_ENREF_38)) | Small bowel perforation (5 weeks following FMT)  Cytomegalovirus infection (7 weeks following FMT)  Hospitalisation for abdominal pain (11 weeks post-FMT)  Cervical carcinoma surgery (6 weeks post FMT) | 1  0  ?  ? | 0  1  ?  ? | X  X  X  X |  |  |
| Moayyedi et al, 2015 ([49](#_ENREF_49)) | Relapse UC requiring colectomy  Patchy inflammation of the colon and rectal abscess  Tested positive for *Clostridium difficile* (7 weeks following FMT) | 0  2  1 | 1  1  0 | X  X  X |  |  |
| Paramsothy et al, 2017([41](#_ENREF_41)) | Deterioration in UC and colectomy  Failure to improve leading to hospitalisation | 1  1 | 0  1 | X  X |  |  |
| Bajaj et al, 2017([32](#_ENREF_32)) | Hospitalisation for acute kidney infection  Hospitalisation for chest pain  Hospitalisation for portal vein thrombosis  Hospitalisation for variceal bleeding  Hepatic encephalopathy  Hospitalisation for diarrhoea  Hospitalisation for anaemia  Hospitalisation for pneumonia  Altered mental status | 1 (84 days post FMT)  1 (114 days post FMT)  0  0  0  0  0  0  0 | 0  1  1  2  5  1  1  1  1 | X  X  X  X  X  X  X  X  X |  |  |
| Bajaj et al, 2019 ([33](#_ENREF_33)) | Hepatic encephalopathy  Death  Hospitalisation due to infection | 1  0  0 | 7  1  3 | X  X  X |  |  |
| Saidani et al, 2019 ([45](#_ENREF_45)) | Death due to pre-existing adenocarcinoma which patient declined treatment for | 1 (3.5 months post FMT) | 0 | X |  |  |
| Huttner et al, 2019 ([51](#_ENREF_51)) | Hospitalization for recurrent UTI  Hospitalization for pyelonephritis  Hepatic encephalopathy in patient with known liver cirrhosis and history of recurrent hepatic encephalopathy  Hospitalisation for fever and abdominal pain  Hospitalization for acute pyelonephritis (pre-existing condition)  Hospitalization for fracture of the proximal femur | 0  0  1 (2 weeks post FMT) -  1 (prior to FMT)  1 (2 weeks post FMT)  1 (4 months post FMT) | 2  1  0 -  0  0  0 | X  X  -  X  X  X | X - |  |
| Sokol et al, 2020 ([46](#_ENREF_46)) | Flare Crohn’s Disease  Gastroenteritis  Food poisoning  Transient asthenia  Cutaneous abscess  Shoulder fracture  Decreased visual acuity | 3  1  1  1  1  0  0 | 6  0  0  0  0  1  1 | X  X  X  X  X |  |  |
| Aroniadis et al, 2019([34](#_ENREF_34)) | Hospitalisation for acute cholecystitis | 0 | 1 | X |  |  |
| Costello et al, 2020 ([42](#_ENREF_42)) | Worsening colitis^$^  *Clostridium difficile* colitis requiring colectomy^$^  Pneumonia^$^ | 1  1  1 | 2  0  0 | ?  ?  ? |  |  |
| Sood et al, 2019 ([52](#_ENREF_52)) | Relapse UC  Death due to myocardial infarction considered unrelated to FMT | 0  1 | 1  0 | X  X |  |  |
| Total reported number of SAE | | 26 | 43 |  |  |  |

$ timing of AE relative to intervention not stated. Likelihood of AE relating to intervention also not stated

**Supplementary Table 5 – Summary of common mild-moderate AE**

| AE | Number of studies reporting on AE (total studies, n=26) | FMT groups | | Control groups | |
| --- | --- | --- | --- | --- | --- |
|  |  | N= | % | N= | % |
| Total number of participants reporting AE | 5 | 95/119 | 80 | 84/116 | 72 |
| Bloating | 6 | 23/121 | 19.0 | 23/118 | 19.5 |
| Nausea | 8 | 51/274 | 18.6 | 26/218 | 11.9 |
| Constipation | 6 | 31/178 | 17.4 | 3/123 | 2.4 |
| Diarrhoea | 7 | 25/149 | 16.8 | 10/150 | 6.7 |
| Abdominal pain | 8 | 47/288 | 16.3 | 38/231 | 16.5 |
| Transient or low grade fever | 5 | 11/131 | 8.4 | 4/133 | 3.0 |
| Vomiting | 6 | 8/136 | 5.9 | 4/136 | 2.9 |

**Supplementary Table 6 – Cochrane Risk of Bias**

| Study | Randomization process | Deviations from intended interventions | Missing outcome data | Measurement of the outcome | Selection of the reported result | Overall Bias |
| --- | --- | --- | --- | --- | --- | --- |
| Tian et al, 2017 ([53](#_ENREF_53)) | \| 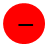 \| \| --- \| | \| 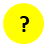 \| \| --- \| | \| 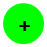 \| \| --- \| | \| 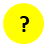 \| \| --- \| | \| 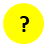 \| \| --- \| | \| 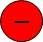 \| \| --- \| |
| Halkjaer et al, 2018 ([56](#_ENREF_56)) | \| 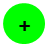 \| \| --- \| | 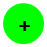 | 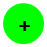 | 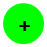 | 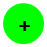 | 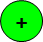 |
| Johnsen et al, 2017 ([44](#_ENREF_44)) | \| 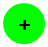 \| \| --- \| | 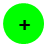 | 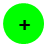 | 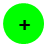 | 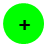 | 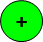 |
| Kootte et al, 2017 ([37](#_ENREF_37)) | \| 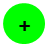 \| \| --- \| | 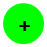 | 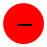 | 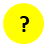 | 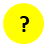 | 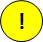 |
| Rossen et al, 2015 ([38](#_ENREF_38)) | \| 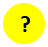 \| \| --- \| | 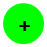 | 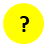 | 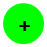 | 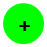 | 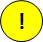 |
| Paramsothy et al, 2017 ([41](#_ENREF_41)) | \| 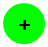 \| \| --- \| | 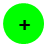 | 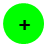 | 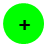 | 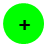 | 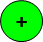 |
| Moayyedi et al, 2015 ([49](#_ENREF_49)) | \| 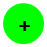 \| \| --- \| | 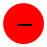 | 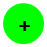 | 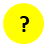 | 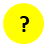 | 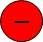 |
| Sokol et al,2020 ([46](#_ENREF_46)) |  |  |  |  |  |  |
| Bajaj et al, 2017 ([32](#_ENREF_32)) |  |  |  |  |  |  |
| Smits et al, 2018 ([39](#_ENREF_39)) |  |  |  |  |  |  |
| Allegretti et al, 2020 ([35](#_ENREF_35)) |  |  |  |  |  |  |
| Holster et al, 2019 ([59](#_ENREF_59)) |  |  |  |  |  |  |
| Vrieze et al, 2012 ([40](#_ENREF_40)) |  |  |  |  |  |  |
| Huttner et al, 2019 ([51](#_ENREF_51)) |  |  |  |  |  |  |
| Herfarth et al, 2019 ([36](#_ENREF_36)) |  |  |  |  |  |  |
| Bajaj et al, 2019 ([33](#_ENREF_33)) |  |  |  |  |  |  |
| Costello et al, 2019 ([42](#_ENREF_42)) |  |  |  |  |  |  |
| Sood et al, 2019 ([52](#_ENREF_52)) |  |  |  |  |  |  |
| Aroniadis et al, 2019 ([34](#_ENREF_34)) |  |  |  |  |  |  |
| El-Salhy et al, 2020 ([43](#_ENREF_43)) |  |  |  |  |  |  |

Low risk , Some concerns, High risk
